# Transient pacing in pigs with complete heart block via myocardial injection of mRNA coding for the T-box transcription factor 18

**DOI:** 10.1038/s41551-024-01211-9

**Published:** 2024-05-02

**Authors:** David W. Wolfson, Nam Kyun Kim, Ki Hong Lee, Jared P. Beyersdorf, Jonathan J. Langberg, Natasha Fernandez, Dahim Choi, Nadine Zureick, Tae Yun Kim, Seongho Bae, Jin-Mo Gu, Jonathan L. Kirschman, Jinqi Fan, Christina Y. Sheng, Danielle Gottlieb Sen, Bret Mettler, Jung Hoon Sung, Young-sup Yoon, Sung-Jin Park, Philip J. Santangelo, Hee Cheol Cho

**Affiliations:** 1 Wallace H. Coulter Department of Biomedical Engineering, Georgia Institute of Technology and Emory University, Atlanta, GA USA; 2 Department of Pediatrics, Emory University School of Medicine, Atlanta, GA USA; 3 Chonnam National University Medical School, Gwangju, South Korea; 4 Division of Cardiology, Department of Medicine, Emory University School of Medicine, Atlanta, GA USA; 5 Division of Pediatric Cardiac Surgery, Department of Surgery, Johns Hopkins School of Medicine, Baltimore, MD USA; 6 Department of Cardiology, CHA Bundang Medical Center, CHA University, Seongnam, South Korea; 7 Blalock-Taussig-Thomas Pediatric and Congenital Heart Center, The Johns Hopkins Children’s Center, Baltimore, MD USA; 8 Department of Biomedical Engineering, Johns Hopkins Whiting School of Engineering, Baltimore, MD USA; 9 Department of Anesthesia and Critical Care Medicine, Johns Hopkins School of Medicine, Baltimore, MD USA

**Keywords:** Gene delivery, Cardiac regeneration, Reprogramming

## Abstract

The adenovirus-mediated somatic transfer of the embryonic T-box transcription factor 18 (*TBX18*) gene can convert chamber cardiomyocytes into induced pacemaker cells. However, the translation of therapeutic TBX18-induced cardiac pacing faces safety challenges. Here we show that the myocardial expression of synthetic TBX18 mRNA in animals generates de novo pacing and limits innate and inflammatory immune responses. In rats, intramyocardially injected mRNA remained localized, whereas direct myocardial injection of an adenovirus carrying a reporter gene resulted in diffuse expression and in substantial spillover to the liver, spleen and lungs. Transient expression of TBX18 mRNA in rats led to de novo automaticity and pacemaker properties and, compared with the injection of adenovirus, to substantial reductions in the expression of inflammatory genes and in activated macrophage populations. In rodent and clinically relevant porcine models of complete heart block, intramyocardially injected TBX18 mRNA provided rate-adaptive cardiac pacing for one month that strongly correlated with the animal’s sinus rhythm and physical activity. TBX18 mRNA may aid the development of biological pacemakers.

## Abstract (web summary)

Intramyocardial injection of synthetic mRNA coding for the embryonic T-box transcription factor 18 gene generates rate-adaptive cardiac pacing and limits innate and inflammatory immune responses, as shown in rodents and pigs.

## Main

Symptomatic bradyarrhythmia is a life-threatening condition if left untreated^1,2^. All current treatments rely on implantation of an electronic pacemaker that mostly consists of a battery-operated generator and electrical lead wires. Although electronic pacemakers generally work well, device-dependent technology suffers from problems inherent to the implanted foreign body as well as device malfunctions^3–5^.

Free from all indwelling hardware, biological pacemakers offer a paradigm-changing approach to cardiac pacing^6^. Immediate clinical needs for biological pacemakers include temporary cardiac pacing where implantable devices fall short. Biological pacemakers could provide a bridge-to-device therapy for patients whose devices are infected^7,8^, display transient conduction disturbances upon aortic valve replacement^9,10^ or suffer from paediatric congenital heart block requiring frequent and invasive surgeries to reposition the pacemaker device^11,12^. For these patients, transient biological pacing would suffice to improve clinical outcomes by offering a device-free interval before definitive device implantation.

We have demonstrated that re-expression of the embryonic T-box transcription factor 18 (TBX18) in the working myocardium creates de novo biological pacemakers in situ^13,14^. We and others have also demonstrated TBX18-induced biological cardiac pacing in a clinically relevant porcine model of heart block^15,16^. However, therapeutic translation of a TBX18 biological pacemaker needs to overcome both unique and common hurdles to gene therapy, which include achieving focal transgene expression, minimizing off-target biodistribution in unintended organs and minimizing immune/inflammatory responses. All previous studies have employed recombinant adenoviral (Adv) vectors for myocardial TBX18 gene transfer^14,15^. The known immune and inflammatory responses elicited by Adv vectors^17–20^, together with its systemic longevity and/or potential inefficacy due to antiviral neutralizing antibodies^21,22^, give pause to the use of viral vectors particularly for the aforementioned indications.

In vitro-transcribed (IVT) synthetic messenger RNA incorporates chemically modified nucleoside analogues to promote translation and limit innate immune responses. This technology has been reported to offer a rapid, dosable gene transfer mode in the heart with substantially reduced innate immune response^23–27^. Earlier efforts to deliver synthetic mRNA in vivo have relied upon cationic lipid carriers, which have potential complications of infusion-related hypersensitivity reactions, tissue injury and local innate immune activation^28–30^. More importantly, lipid-based delivery systems would increase the risk of off-target transgene expression, such as to the liver and spleen, rather than remaining focal to the injection site in the heart. Recent evidence suggested that delivery of mRNA to the heart without a transfection reagent resulted in expression of a reporter transgene in vivo^31^. We hypothesized that direct gene transfer of unformulated or ‘naked’ IVT TBX18 mRNA creates de novo ventricular pacing in vivo while avoiding overt immune responses or off-target biodistribution.

In this Article, we investigated the efficiency of synthetic IVT mRNA-based gene transfer to cardiomyocytes in vitro and in vivo, and then compared and contrasted the mRNA-based gene transfer with Adv vector-mediated transduction. Our data draw clear distinctions in the expression kinetics, focal versus diffuse expression, and innate immune responses of the two gene transfer modalities. Delivery of TBX18 mRNA at the left ventricular (LV) apex of rodents significantly increased the frequency of ectopic heartbeats near the injection site. Importantly, TBX18 mRNA showed biological pacing function and chronotropic competence in a clinically relevant large animal model of complete atrioventricular block (CAVB) over a 4-week study period. Our data demonstrate bioengineering of a non-viral gene therapy for a biological pacemaker with efficacy and safety for temporary heart rate control.

## Results

### Rapid and transient transgene expression by synthetic mRNA

**Fig. 1:**
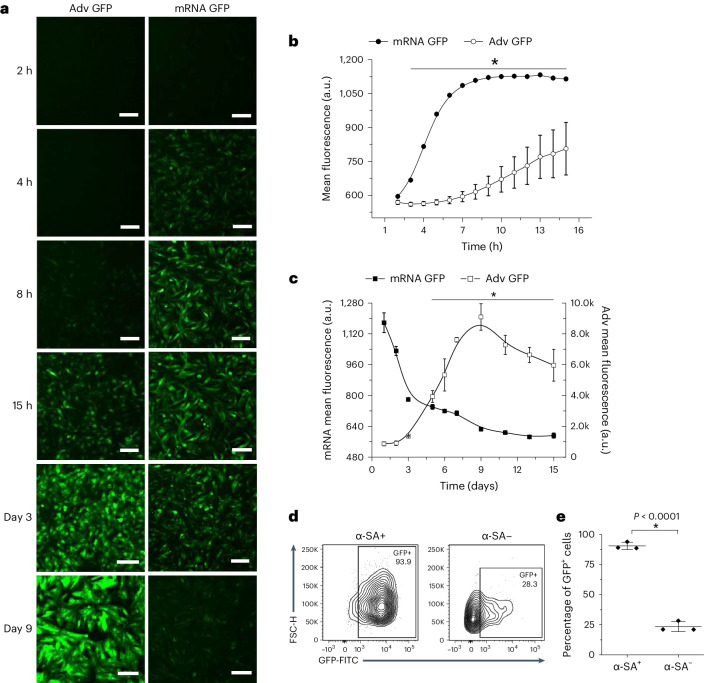
Myocardial gene transfer of synthetic mRNA is rapid and transient. **a**, Representative time-lapse GFP fluorescence images from Adv GFP- or mRNA GFP-treated NRVMs (*n* = 3 wells each). Scale bars, 150 μm. **b**,**c**, The mean GFP fluorescence of mRNA GFP (*n* = 3 wells) and Adv GFP (*n* = 3 wells) during the first 15 h (**b**) and 15 days (**c**) post-gene transfer. Mean ± s.d. *P < 0.05, temporal comparisons with two-way repeated measures ANOVA with Tukey’s test. **d**, Representative flow cytometry contour of GFP expression in α-SA^+/−^ cells 1 day post-transfection with mRNA GFP (*n* = 3 wells). Each well was sorted between α-SA^+^ cardiomyocytes (left) and α-SA^−^ non-myocytes (right), before GFP gating. **e**, The proportion of GFP^+^ cells was significantly higher in myocytes versus non-myocytes (*n* = 3 wells). Mean ± s.d. *P < 0.05, two-sided two-sample *t*-test. FSC, forward scatter; FITC, fluorescein isothiocyanate.

Synthetic mRNA constructs were in vitro transcribed on the basis of previously published methods^24,27,32–34^. To understand the gene expression kinetics, neonatal rat ventricular myocytes (NRVMs) were transduced with adenovirus or transfected with IVT mRNA encoding green fluorescent protein (GFP) (Adv GFP and mRNA GFP, respectively) (Fig. 1a). Time-lapse microscopy revealed robust GFP expression as early as 4 h post-transfection of mRNA GFP, whereas NRVMs transduced with Adv GFP did not show significant GFP fluorescence until 10 h post-transduction (Fig. 1a,b and Supplementary Video 1). Fluorescence began to decay in the days following mRNA GFP’s peak during the first 24 h, while Adv GFP-treated NRVMs’ fluorescence continued to increase linearly up to 7 days post-transduction (Fig. 1c).

Non-myocytes such as fibroblasts and endothelial cells are substantial constituents of the myocardium^35–40^. We asked whether there were differences in transfection efficiency of synthetic mRNA between α-sarcomeric actinin (α-SA)-expressing cardiomyocytes and non-myocytes in our NRVM cultures. Flow cytometry of the NRVM monolayers indicated that about 30% of the population are non-myocytes as gated by α-SA staining (Supplementary Fig. 1a,b). Transfected NRVM monolayers revealed 90.5 ± 1.7% of α-SA^+^ cardiomyocytes were GFP^+^ on day 1. In contrast, 25% of α-SA^−^ non-myocytes were GFP^+^ by day 1 (Fig. 1c). The dose of mRNA affected transfection efficiency as well. At a dose of 0.1 ng per 1,000 cells, few cells were GFP^+^ on day 1 (Supplementary Fig. 1d). Above 1 ng per 1,000 cells, the percentage of GFP^+^ cells and mean GFP fluorescence did not increase for either cardiomyocytes or non-myocytes (Supplementary Fig. 1d,e). The data establish an upper limit for the dosing of synthetic mRNA transfection in primary cardiac myocytes at 1 pg per cell.

### TBX18 mRNA reprogrammes cardiomyocytes to pacemaker cells

**Extended Data Fig. 1:**
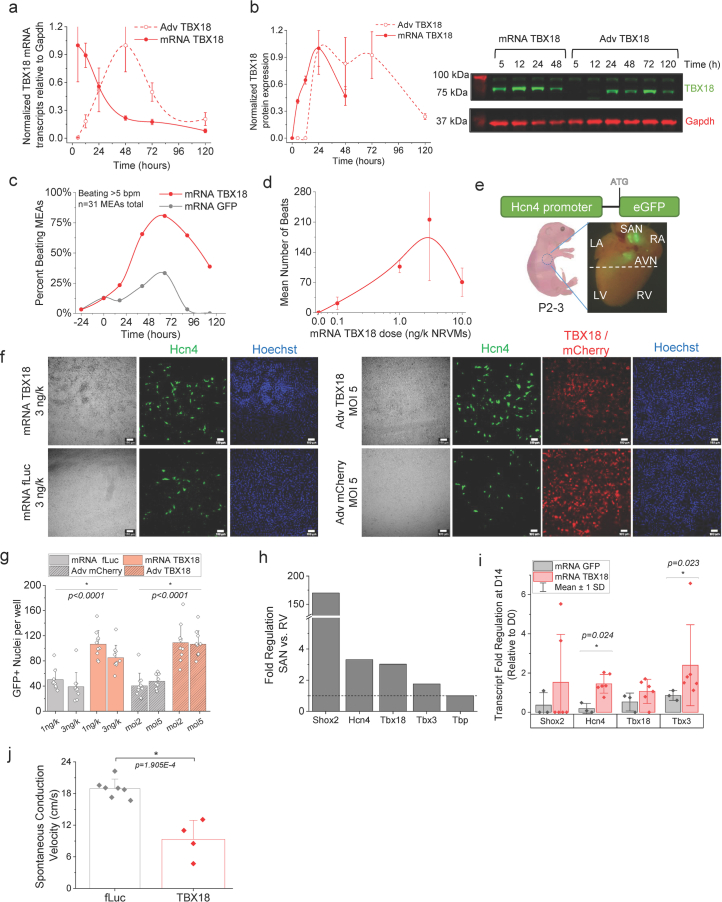
In vitro functional characterization of TBX18 mRNA^−^derived pacemaker cells. **a**, Normalized TBX18 mRNA transcript level relative to Gapdh in NRVMs treated with Adv TBX18 or mRNA TBX18 (n = 4 wells, Mean + /-SD). **b**, Normalized TBX18 protein expression relative to Gapdh in NRVMs post-gene transfer (n = 4 wells, Mean + /-SEM). Quantified TBX18 expression was normalized to peak TBX18 expression for each vector. Representative Western Blot stained for TBX18 (green) and Gapdh (red) at time (hours) post-gene transfer. **c**, Percent of beating wells for mRNA TBX18- or GFP-transfected NRVMs. Beating MEA defined as averaging >5 bpm over a 30-minute recording. **d**, Mean number of beats per MEA in NRVMs transfected with various doses of IVT TBX18 (n = 9 MEAs, Mean + /-SEM) at 43-hours post-transfection. **e**, Transgenic scheme of Hcn4/GFP mice. SAN and AVN, expressing Hcn4, can be visualized with GFP. Only ventricles below the AVN were collected for isolation and culture. **f**, Representative fluorescence images from Hcn4/GFP Tg NMVMs transfected with 3 ng/k cells of mRNA (left), or transduced with Adv multiplicity of infection of 5 (right). Scale bar, 100 µm. Experiment repeated independently twice. **g**, Quantified number of GFP-expressing cells was higher in TBX18 NMVMs, compared to respective control. (n = 10 wells, Mean + /-SD, *p < 0.05 2-way ANOVA with Tukey’s test, See Supplementary Tables 3-4 for p value results). **h**, **i**, Relative expression levels of pacemaker-enriched genes found in isolated neonatal rat SAN tissues (n = 1 samples with 20 pooled independent tissues) (**H**) and mRNA TBX18-transfected NRVMs 14-days post transfection (n = 3 wells GFP, n = 6 wells TBX18, *p < 0.05 two-sided Mann-Whitney Test) (**I**). **j**, Measured conduction velocity of spontaneous beat propagations in transfected NRVM monolayers (n = 7 wells fLuc, n = 4 wells TBX18, Mean + /-SD, *p < 0.05 1-way ANOVA with Tukey’s test). Source data

Transfection of synthetic IVT mRNA encoding TBX18 into NRVMs led to rapid uptake of mRNA TBX18 transcripts leading to its peak intracellular level in <5 h, but only about 20% of the peak level remained by day 2 (Extended Data Fig. 1a). In contrast, it took 2 days for Adv TBX18 to reach its peak transcript levels and 5 days to reach 20% of the peak level (Extended Data Fig. 1a). Translated TBX18 protein products followed their respective transcript levels. TBX18 protein translated from IVT mRNA was robustly expressed as early as 5 h post-transfection and peaked at 24 h post-transfection. TBX18 protein translated from Adv was not detectable until 24 h post-transduction and remained elevated up to 72 h thereafter (Extended Data Fig. 1b).

**Fig. 2:**
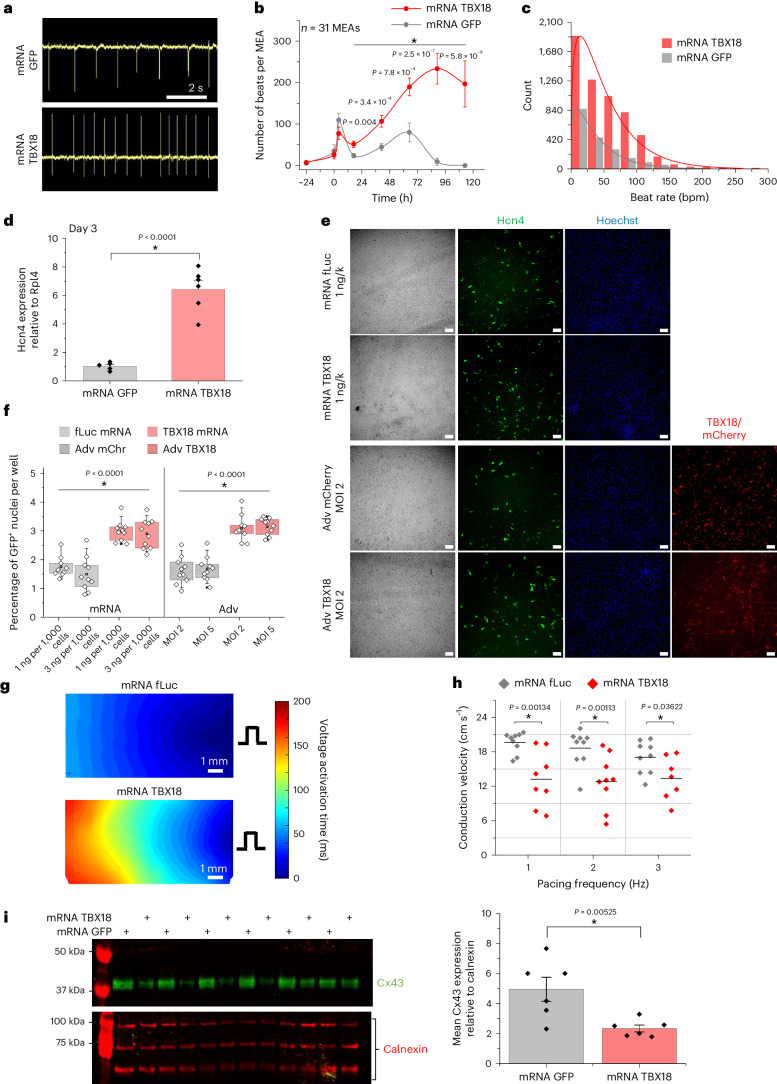
TBX18 mRNA reprogrammes primary ventricular myocytes to pacemaker cells. **a**, A representative 8 s recording of extracellular potential changes with MEA (*n* = 31 MEAs). Scale bar, 2 s. **b**,**c**, Spontaneous beats recorded per MEA (**b**) and beat rate frequencies at day 3 (**c**) were significantly higher for mRNA TBX18-transfected NRVMs over GFP in the days following transfection (*n* = 31 MEAs per group, mean ± s.e.m., *P < 0.05, two-sided *t*-test comparison GFP versus TBX18 at each timepoint). **d**, Quantitative PCR of Hcn4 transcript levels was significantly higher in mRNA TBX18-transfected compared with mRNA GFP-transfected NRVMs on D3 post-transfection (*n* = 6 wells, mean ± s.e.m., *P < 0.05, one-sided two-sample *t*-test). **e**, Representative fluorescence images from Hcn4/GFP Tg NMVMs transfected with mRNA fLuc or mRNA TBX18 (top), or transduced with Adv mCherry or Adv TBX18-DsRed (bottom). Scale bars, 100 µm. The experiment was repeated independently twice. **f**, The mean proportion of Hcn4/GFP-expressing nuclei was significantly higher for both mRNA TBX18 and Adv TBX18, compared with their respective controls. *n* = 10 wells. *P < 0.05, two-way ANOVA with Tukey’s test (*P* values reported in Supplementary Tables 1 and 2). Box plot, 25th–75th percentiles; centre, mean; whisker, 10th–90th percentiles. No differences observed between doses. **g**, Representative colour contour plots of voltage activation time across NRVM monolayers transfected with mRNA fLuc (left) or mRNA TBX18 (right). **h**, The mean conduction velocity measured from contour maps at various pacing frequencies was significantly lower in mRNA TBX18-transfected monolayers (*n* = 8) compared with mRNA fLuc (*n* = 9). *P < 0.05, two-way repeated measures ANOVA with Tukey’s test. **i**, Immunoblotting revealed significantly lower Cx43 protein in mRNA TBX18-transfected NRVMs, compared with GFP (alternating wells, *n* = 6 wells, *P < 0.05, one-sided two-sample *t*-test). Mean ± s.d. DsRed is a red fluorescent protein from a corallimorpharian of the *Discosoma* genus. Source data

Somatic cell reprogramming does not require persistent expression of the exogenous reprogramming factors after arriving at the final cell fate^41–43^. We reasoned that brief expression of TBX18 mRNA suffices to convert chamber cardiomyocytes to induced pacemaker cells as has been demonstrated with Adv vectors^13–15^. To test whether the transient expression window of mRNA TBX18 sufficed to induce automaticity in NRVMs, we examined spontaneous oscillations of extracellular field potentials from NRVM monolayers cultured on multielectrode arrays (MEAs; Fig. 2a). Before transfection, both groups of MEAs showed similar degrees of spontaneity (Fig. 2b). TBX18-transfected NRVMs produced significantly more spontaneous beats per well, compared with the mRNA GFP control (Fig. 2a,b). Additionally, TBX18-transfected NRVMs exhibited a significantly higher number of MEAs with spontaneous electrical activity compared with mRNA GFP-transfected NRVMs (25/31 versus 10/30 wells, respectively), starting at least 12 h post-transfection, and lasting over the 1-week culture period (Extended Data Fig. 1c). At 3 days post-transfection, beat rate histograms revealed that NRVMs transfected with mRNA TBX18 showed a significantly higher beat distribution across all frequencies (Fig. 2c). The dose of TBX18 mRNA necessary to induce automaticity was between 1 and 3 ng per 1,000 cells NRVMs with a slight decrease at the highest dose (10 ng per 1,000 cells) (Extended Data Fig. 1d). This decrease in spontaneity may be due to cytotoxic effects of the transfection reagent, mRNA or both.

**Extended Data Fig. 2:**
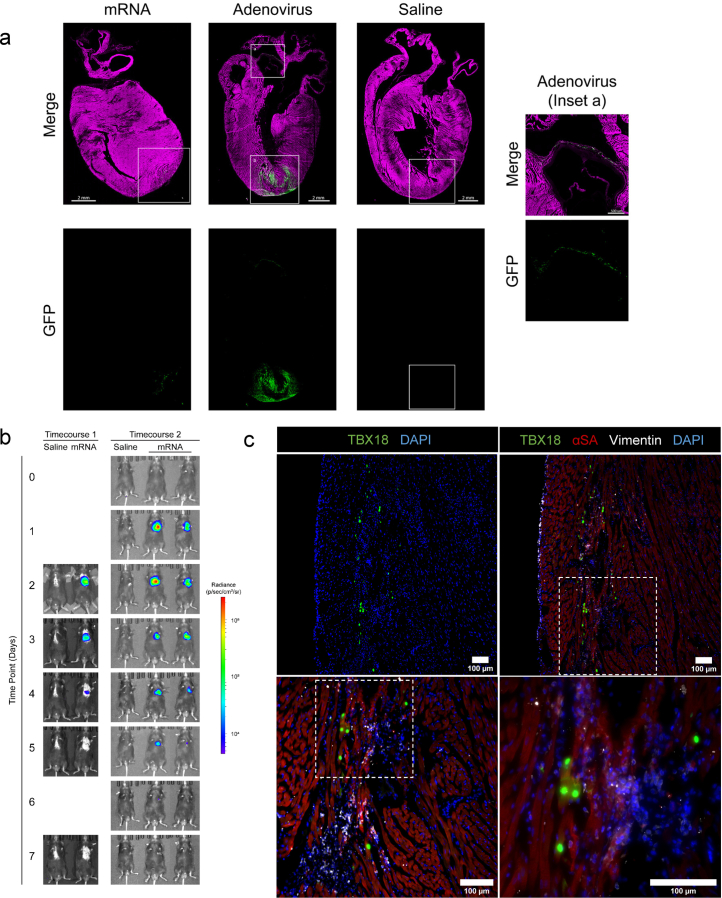
IVT mRNA transfection in vivo by direct myocardial injection. **a**, Immunostained images of hearts injected with either GFP mRNA (left), Adv (middle), or saline. Heart sections were co-stained for alpha-sarcomeric actinin (magenta, top). Focal GFP expression was observed in mRNA injected. Adv GFP injected showed widespread GFP fluorescence throughout the chamber wall with additional off-target fluorescence in the endothelial wall of the aorta (inset a). Experiment repeated independently twice. **b**, Temporal IVIS imaging of mice injected with either saline (left) or fLuc mRNA (right). Color maps represent intensity of detected bioluminescence signal (scale bar, right). These results were observed in 2 independent experiments (labeled top). **c**, Successful transfection of TBX18 mRNA in rat hearts, showing nuclear TBX18 expression in aSA+ cardiomyocytes. Dashed box, magnified area of interest. Experiment repeated independently twice.

Spontaneously beating cells of the cardiac conduction system are routinely identified by the ion channel, hyperpolarization-activated cyclic nucleotide-gated potassium channel 4 (*Hcn4*), one of the major components for generating spontaneous phase 4 depolarization^44^. Corresponding with the increased spontaneity observed with MEA (Fig. 2b), TBX18-transfected NRVMs contained >6-fold higher *Hcn4* transcript level compared with control at day 3 post-transfection (Fig. 2d; *n* = 6 wells). We employed a transgenic mouse line (Mouse Genome Informatics: 4847123), containing a bacterial artificial chromosome with the enhanced GFP (eGFP) reporter gene at the first coding exon of the *Hcn4* gene (*Hcn4*^GFP/+^). *Hcn4*^GFP/+^ neonatal mouse ventricular myocytes (NMVMs) were isolated and cultured (Extended Data Fig. 1e). Twelve days after in vitro gene transfer, live-cell imaging of Hoechst-stained NMVMs revealed a significantly higher proportion of GFP^+^ myocytes in both mRNA TBX18- and Adv TBX18-treated wells, compared with controls mRNA firefly luciferase (fLuc) or Adv mCherry (Fig. 2e,f; *n* = 10 wells). The proportion of GFP^+^ cells out of total nuclei was similar in both TBX18-treated groups (mRNA and Adv), indicating *Hcn4* expression is comparable between mRNA and Adv vectors (Fig. 2f). Additionally, NMVMs treated with a higher dose of each TBX18 vector (3 ng per 1,000 cells mRNA, multiplicity of infection (MOI) 5 Adv) showed a significantly higher number of GFP^+^ myocytes compared with respective controls, yet were similar to NMVMs treated with lower doses of mRNA TBX18 or Adv TBX18 (Extended Data Fig. 2f,g). Further, we found the sinoatrial node (SAN) pacemaker-enriched transcripts, *Hcn4* and *Tbx3* (Extended Data Fig. 1h), were significantly higher in TBX18-transfected NRVMs compared with GFP at 2 weeks post-transfection (Extended Data Fig. 1i; *n* = 6 wells). No statistical difference was found in *Shox2* and *Tbx18* transcript level (Extended Data Fig. 1i). Combined with <5 days of TBX18 protein life (Extended Data Fig. 1b), these data indicate *Hcn4* expression lasting beyond the transient expression window of TBX18 mRNA.

Electrical conduction within the cardiac pacemaker tissue is characterized by a collection of weakly coupled pacemaker and non-pacemaker cells that have a relatively slow electrical propagation velocity compared with the general myocardium, which protects itself from the hyperpolarizing atrial myocardium^45,46^. NRVM monolayers transfected with either mRNA TBX18 or fLuc were loaded with the voltage-sensitive dye FluoVolt (Invitrogen) to optically map electrical propagation patterns at 2–3 days post-transfection (Fig. 2g and Supplementary Videos 2 and 3). Spontaneous propagations showed significantly lower conduction velocities in TBX18-transfected monolayers compared with fLuc (Extended Data Fig. 1j). Similarly, when paced, TBX18-transfected monolayers presented significantly slower conduction compared with fLuc for all frequencies (Fig. 2h; *n* = 8 samples). Pacing at higher frequencies decreased the mean conduction velocity of fLuc-transfected monolayers, but not in TBX18 (Fig. 2h). This is in line with the inverse relationship between pacing frequency and conduction velocity^47^, probably due to the inactivation of Na^+^ channels in the fLuc monolayers^48^. We have previously shown that overexpression of Adv TBX18 in NRVMs lead to suppression of the predominantly myocardial gap junction protein, Cx43 (ref. ^13^). In line with our previous findings, mRNA TBX18-transfected NRVMs exhibited significantly lower levels of Cx43 protein compared with GFP (Fig. 2i; *n* = 6 wells), probably accounting for the slower conduction observed in TBX18. Together, mRNA TBX18 gene transfer in cardiomyocytes led to increased automaticity and *Hcn4* expression and slower conduction with lower Cx43 expression, recapitulating key electrophysiological properties of native SAN pacemaker cells^45,46,49^.

### Myocardial transfection of naked mRNA in vivo

**Fig. 3:**
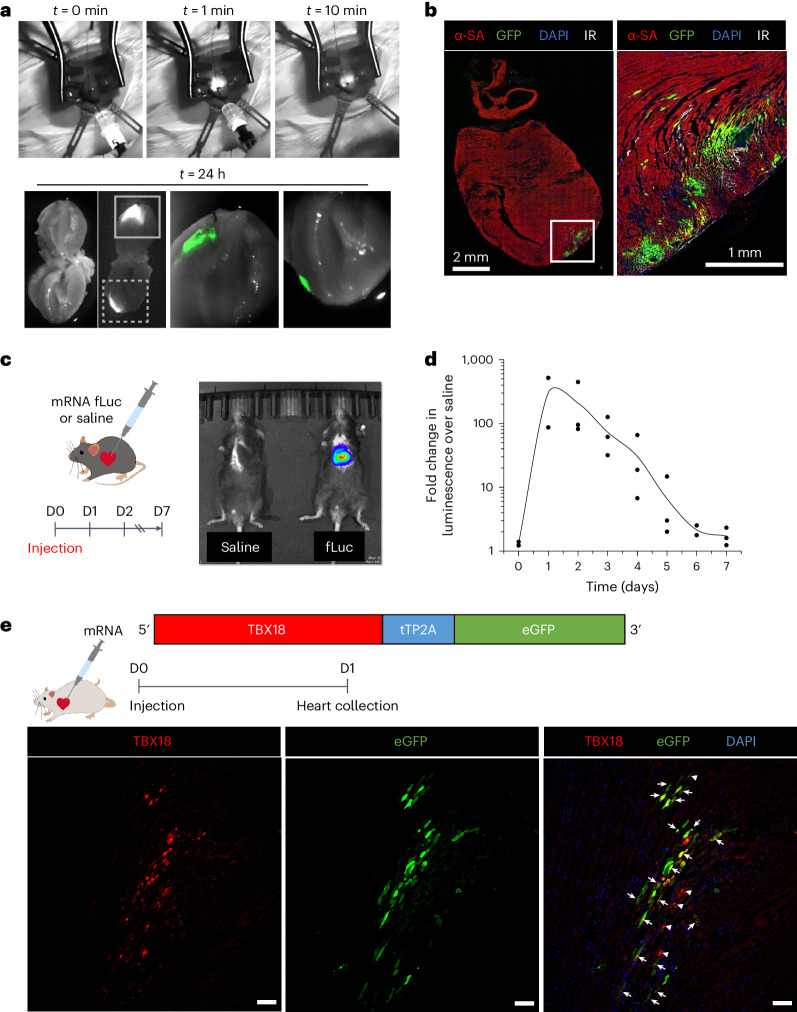
Myocardial transfection of naked mRNA in vivo. **a**, Injection of Dylight-tagged GFP mRNA at the rat LV apex. The injection site was observed for 10 min to confirm tissue retention. Twenty-four hours post-injection, the heart was cut via the frontal plane. Solid and dashed magnified insets (bottom right) show GFP fluorescence overlap with areas of Dylight mRNA. **b**, Immunostained GFP^+^ myocytes at the injection site 1 day post mRNA GFP injection (left) with magnified inset (right). The experiment was repeated independently twice. **c**, Mice injected with either mRNA fLuc or saline at the LV apex were imaged daily with IVIS. mRNA fLuc-injected mice showed bioluminescence signal localized to the thoracic cage, 1 day following injection. D, day. **d**, Daily bioluminescence normalized to saline-injected mice shows transient expression of mRNA fLuc for <7 days (*n* = 3 mice). **e**, Myocardial injection of naked TBX18-tTP2A-eGFP mRNA leads to expression of TBX18 with GFP reporter in successfully transfected cells in rats. The experiment was repeated independently twice (arrows, TBX18 and GFP expression in same cell; arrowheads, TBX18 expression without GFP). DAPI, 4′,6-diamidino-2-phenylindole.

Upon validating in vitro gene transfer of synthetic mRNA into primary ventricular cardiomyocytes, we sought to characterize in vivo gene transfer of naked, synthetic mRNA dissolved in RNase-free saline via direct myocardial injection. To visualize the mRNA solution during delivery, IVT GFP mRNA was annealed with RNA probes conjugated to a near-infrared (IR) dye (Dylight 680-NHS esters, ThermoFisher). This dye could be visualized in real time with an IR camera (Fig. 3a and Supplementary Videos 4 and 5). Twenty-four hours post-injection, the IR dye could still be visualized in the myocardium with GFP^+^ expression directly overlying the injection site (Fig. 3a). GFP fluorescence was strictly focal to the injection site at the LV apex (Fig. 3b). In contrast, rats injected with Adv GFP showed broad transgene expression through the LV wall and reaching endothelium of the aorta (Extended Data Fig. 2a).

To understand the kinetics of in vivo protein expression via IVT mRNA, a transgene encoding fLuc was generated as mRNA or Adv. We examined the fLuc bioluminescence in a longitudinal manner for 7 days post gene injection using an in vivo imaging system (IVIS; Fig. 3c and Extended Data Fig. 2b). Mice injected with mRNA fLuc (150 µg, *n* = 3) at the LV apex showed robust bioluminescence localized at the cardiothoracic region, which peaked at 24 h and decayed exponentially over 6 days post-gene transfer (Fig. 3d).

Naked TBX18 mRNA was also confirmed to be successfully transfected in cardiomyocytes via direct myocardial injection in rats. Co-staining with TBX18, α-SA and vimentin showed that the vast majority of TBX18^+^ nuclei localized to α-SA^+^ myocytes at the site of injection (Extended Data Fig. 2c). Additionally, we found that TBX18 mRNA can be transfected to the myocardium as a cistronic vector with eGFP by incorporating a tTP2A self-cleavable peptide (Fig. 3e). A majority of TBX18^+^ nuclei co-expressed eGFP (Fig. 3e, arrows) with a minority expressing TBX18 alone (Fig. 3e, arrowheads).

### Myocardial in vivo mRNA delivery is minimally immunogenic

**Fig. 4:**
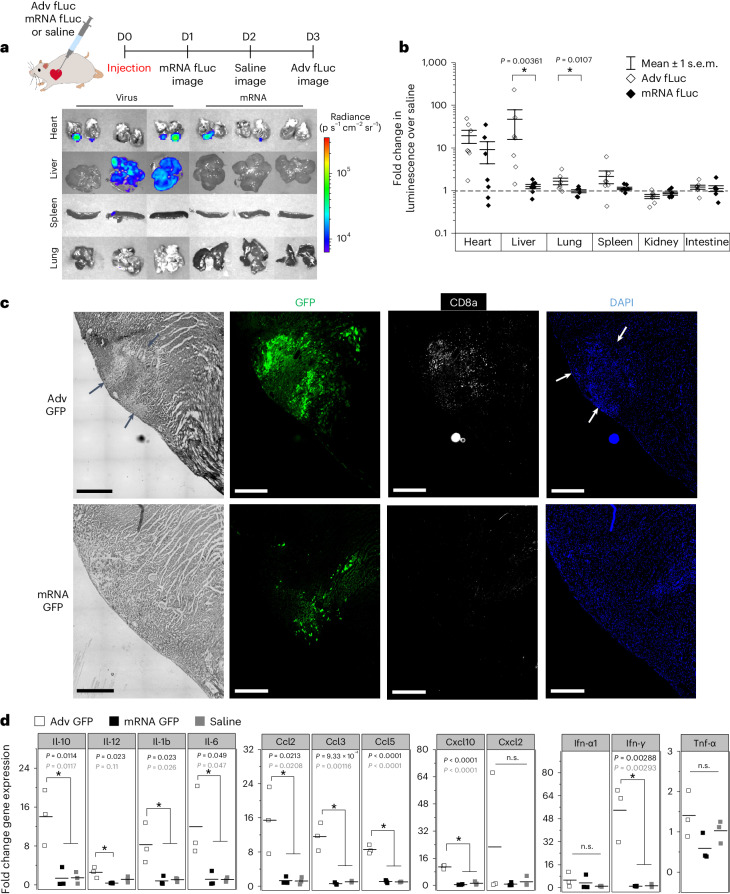
Naked myocardial mRNA delivery is focal, with minimal immune responses in vivo. **a**, Representative IVIS images of isolated organs from Adv fLuc- (left) and mRNA fLuc- (right) injected rats. D, day. **b**, The saline-normalized luminescence flux was significantly higher in off-target organs of Adv fLuc-injected rats (*n* = 7). Box, mean ± s.e.m. Dashed line, saline. *P < 0.05, one-sided Mann–Whitney *U* test. **c**, Representative images of Adv GFP- and mRNA GFP-injected hearts at LV apex. CD8a-immunostained (white) infiltrating leukocytes surrounded GFP-expressing cells. Black arrows, areas of changed tissue morphology. White arrows, areas of high-density nuclei. Scale bars, 500 μm. The experiment was repeated independently twice. **d**, A quantitative PCR panel of fold change gene expression for select cytokines, chemokines and interferons in hearts 7 days post-injection of Adv GFP, mRNA GFP or saline (*n* = 3). *P < 0.05, one-way ANOVA with Tukey’s test. Top *P* value, Adv GFP versus mRNA GFP. Bottom *P* value, Adv GFP versus saline. n.s., not significant.

To better understand off-target gene expression with mRNA compared with viral vectors, rats were injected with either mRNA fLuc (300 µg), Adv fLuc (0.5 × 10^9^ plaque-forming units (PFU)) or saline at the LV myocardium in a volume of 100 µl (Fig. 4a). Animals injected with Adv fLuc showed significant off-target bioluminescence in the liver and lungs in addition to the intended organ, the heart (Fig. 4a,b and Supplementary Fig. 2a). In animals where myocardial gene delivery was compromised, fLuc expression in the liver was particularly intense (Fig. 4a, virus), indicating that failed myocardial gene delivery may lead to unintended consequences with Adv vectors. In contrast, rats injected with fLuc mRNA showed bioluminescence exclusively in the heart with no off-target expression, including cases of failed transfection (*n* = 7 rats) (Fig. 4a,b). This suggests that unintended delivery of naked mRNA to systemic circulation may be labile and unable to induce global gene transfer in other organs. The average luminescence flux at the heart was statistically similar between mRNA fLuc- and Adv fLuc-injected myocardium, though variability in successful transgene expression is increased with mRNA (Fig. 4b).

One of the advantages of synthetic IVT mRNA is its restrained immune response compared with viral vectors. To test for this, adult rats were injected at the LV apex with either mRNA GFP (300 µg), Adv GFP (0.5 × 10^9^ PFU) or equivolume saline. To understand the types and quantities of immune modulatory cells infiltrating the gene delivery site, the hearts were collected and immunostained at the injection site for pan-leukocyte markers, CD8a, CD11b/c and CD45 (Fig. 4c and Supplementary Fig. 2b–d). Cytotoxic T lymphocytes (CD8a^+^ cells) were detected at a considerably higher density in the myocardium injected with Adv GFP compared with those injected with mRNA GFP (Fig. 4c and Supplementary Fig. 2b). This was accompanied by a densely nucleated region at the Adv GFP injection site (white arrows) but not in mRNA GFP injection site, suggesting homing of other immune modulatory cells. Supporting this notion, Adv GFP-injected hearts exhibited larger populations of CD11b/c (cardiac macrophages; Supplementary Fig. 2c) and CD45 (pan-leukocyte; Supplementary Fig. 2d) cells proximal to GFP-expressing myocytes compared with mRNA GFP-injected hearts.

Key to immune activation is the induction of inflammatory cytokines. We measured transcript levels of cytokines and chemokines in the whole heart at 1 week post-injection, using a panel of acute inflammatory interleukins, chemokines and interferons. The expression levels of most interleukins (Il-10, Il-12, Il-1b and Il-6), chemokines (Ccl2, Ccl3, Ccl5 and Cxcl10) and interferon (Ifn-γ) were substantially higher in Adv GFP compared with those in mRNA GFP-injected hearts (Fig. 4d). Indeed, mRNA GFP-injected hearts showed no difference in gene expression of all measured genes relative to saline-injected controls (Fig. 4d).

### TBX18 mRNA injection leads to de novo cardiac pacing in rats

**Fig. 5:**
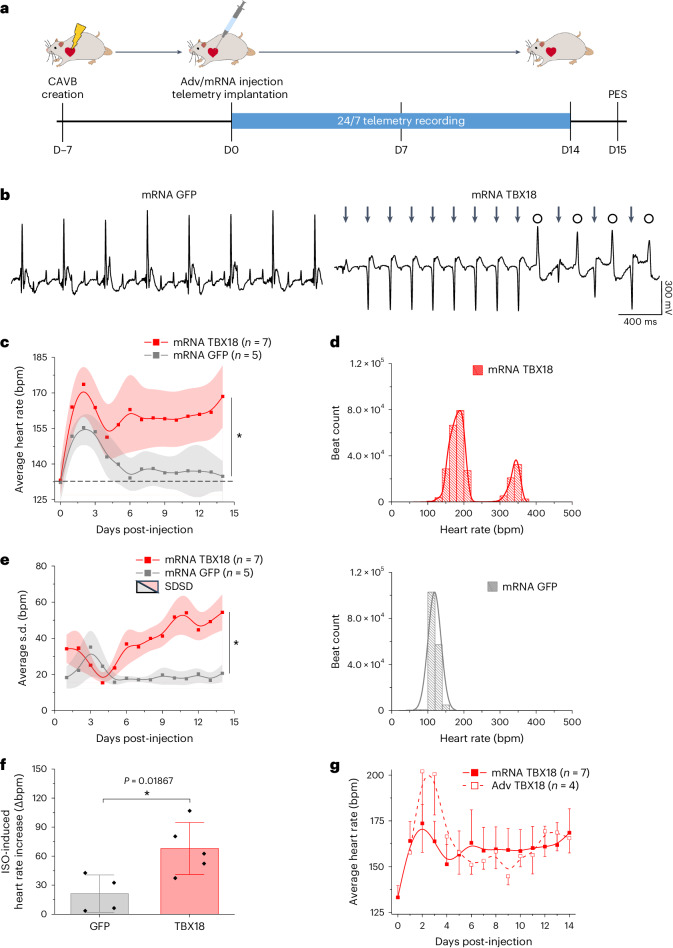
TBX18 mRNA-induced ventricular pacing in a small animal model of CAVB. **a**, Schematic of in vivo study design. **b**, A representative telemetry ECG strip of mRNA GFP (left) or mRNA TBX18 (right) rats (*n* = 7 rats TBX18, *n* = 5 rats GFP). Arrows, fast negative polarity beats; circles, slow junctional escape rhythm. **c**, Daily mean heart rate ± s.e.m was higher for mRNA TBX18 over 2 weeks post-injection. Dashed line, average baseline for all rats before mRNA injection. Mean centre point ± s.e.m. *P < 0.05, two-way ANOVA repeated measures with Tukey’s test. **d**, Representative beat rate histograms for mRNA TBX18 (top) and GFP (bottom) rats on day (D)7. **e**, The average heart rate s.d. ± standard deviation of s.d. (SDSD) was higher for mRNA TBX18 over GFP. Error bands represent s.d. *P < 0.05, two-way ANOVA repeated measures with Tukey’s test. **f**, TBX18-treated rats showed significantly higher ISO-induced (3 mg kg^−1^, i.p.) increases in heart rate. *P < 0.05, one-sided Mann–Whitney *U* test. *n* = 5 TBX18 rats, *n* = 4 GFP. Average ± s.d. recorded from surface ECG. **g**, Daily average heart rate ± s.e.m. comparison between CAVB rats treated with mRNA TBX18 (solid) or Adv TBX18 (dashed).

To test whether an mRNA-based gene therapy could achieve disease-modifying activity for a severe bradyarrhythmia, we took advantage of our rat model of chronic CAVB^50^. Upon model creation, animals were monitored for 1 week to confirm stable CAVB. Confirmed heart block rats were injected at the LV apex with either TBX18 mRNA (300 µg, *n* = 7) or GFP mRNA (300 µg, *n* = 5), and were implanted with a telemeter for continuous recording of ambulatory electrocardiogram (ECG) over 2 weeks (Fig. 5a). The LV apex was a deliberate site of TBX18 injection since pacing from the apex is readily discernible with negative QRS complexes in lead II due to retrograde conduction. By day14, control animals were supported by slow junctional escape beats at ~130 bpm (Fig. 5b, left). In contrast, TBX18-injected animals exhibited frequent runs of QRS complexes that were faster and negative in polarity (Fig. 5b, right, arrows), which appeared to compete with the slow junctional escape rhythm (circles). The negative polarity of QRS complexes in TBX18-injected animals indicates retrograde conduction in line with the LV apex injection site.

**Extended Data Fig. 3:**
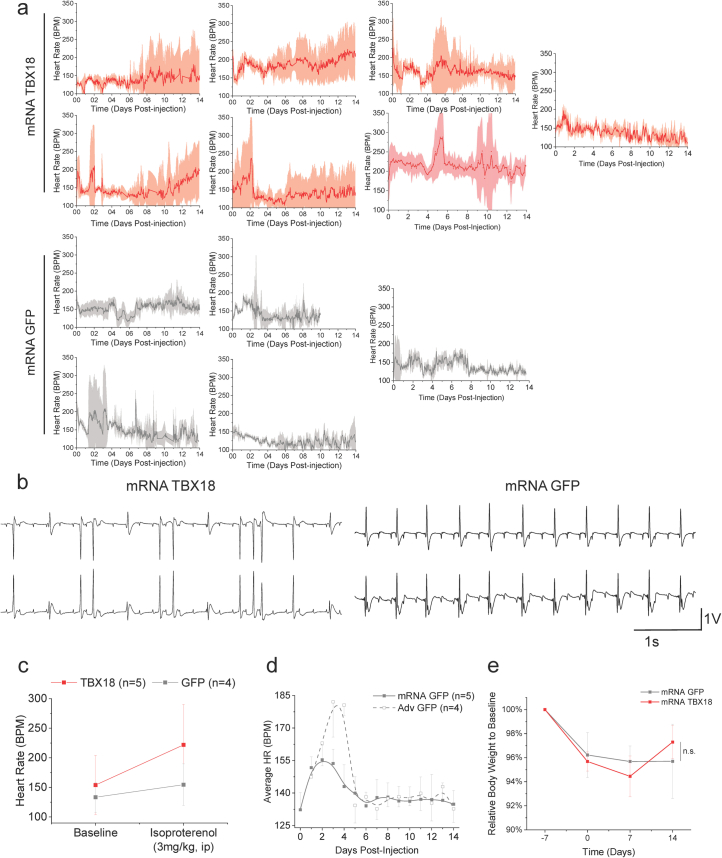
Functional TBX18 IVT mRNA induced-biological pacing in vivo. **a**, IVT TBX18- (top) or IVT GFP- (bottom) injected rats, ECG telemetry analyzed for 1-hour averaged heart rate +/- standard deviation plotted over the 2-week recording period for each rat (n = 7 TBX18, n = 5 GFP). **b**, Representative ECG telemetry strip of an IVT TBX18 (left) and GFP (right) rat at Day 14, when heart rate variability has significantly increased in TBX18-treated rats. Lead 1, top. Lead 2, bottom. **c**, Heart rate of TBX18 and GFP treated rats under anesthesia (baseline) and after bolus injection of isoproterenol at D14 (n = 4 rats GFP, n = 5 rats TBX18, Mean + /-SD). **d**, Daily average heart rate measured from ECG telemetry of rats treated with either GFP Adv or mRNA (n = 5 rats GFP mRNA, n = 4 rats GFP Adv, Mean + /-SEM). **e**, Average body weight relative to baseline +/- SEM for TBX18 (red) and GFP (gray) rats plotted at each time-point along the study (n = 5 rats TBX18, n = 4 rats GFP). *p < 0.05 2-way ANOVA repeated measures with subsequent Tukey’s test.

One-hour average heart rates were recorded for each animal over the 2-week study period (Extended Data Fig. 3a). During the first 3 days following gene delivery, heart rates from all animals increased, probably due to acute inflammation from the second thoracotomy for gene delivery. By day 6, control animals’ mean heart rate returned to the baseline and remained slow. In contrast, TBX18-injected animals’ mean heart rate was significantly higher throughout the 2-week study period (Fig. 5c). To examine whether competing pacemaker foci could be revealed, ventricular rate histograms were plotted for a 24-h period at day 7 (Fig. 5d). TBX18-injected animals manifested two major peaks, one at 181 bpm and the other at 343 bpm, whereas GFP-injected animals showed a single peak at 130 bpm (Fig. 5d). The faster ventricular rate of TBX18 mRNA-injected animals replicated the animals’ normal sinus rhythm of 320–380 bpm (ref. ^51^). During the slower ventricular events from the 181 bpm histogram peak, we observed a lower frequency of competing ventricular beats in TBX18 rats (Extended Data Fig. 3b). From these heart rate histograms, the overall persistence of TBX18-induced beats can be assessed. Being injected outside of the normal conduction system (LV apex), we expected that the de novo pacemaker site may encounter issues of source–sink mismatch that hinder the ability to consistently pace at a steady rate. The standard deviation (s.d.) of the mean heart rates grew greater over time in TBX18-injected rats, but not in control over 2 weeks (Fig. 5e).

Natural sinus rhythm readily rate-adapts to autonomic inputs. Our continuous heart rate telemetry data illustrated greater variabilities in the mean heart rate over time (Fig. 5c,e), which may correlate to responses to physiological needs. To understand capacity of TBX18 mRNA-induced biopacing to rate-adapt, the animals were sedated and subjected to β-adrenergic stimulation via isoproterenol (ISO). Upon injection of ISO (3 mg kg^−1^, intraperitoneal (i.p.)), the mean heart rate increased by 44% to 222 ± 68 bpm in mRNA TBX18-injected animals compared with 16% to 154 ± 35 bpm in GFP-injected animals (Extended Data Fig. 3c). This illustrated a notably higher capacity to rate-adapt in mRNA TBX18-injected animals compared with control (68 ± 27 versus 21 ± 19 bpm, respectively, *n* = 5 TBX18, *n* = 4 GFP; Fig. 5f).

Motivated by our data that induction of *Hcn4* was comparable between mRNA and Adv gene transfer of TBX18 in vitro (Fig. 2e,f), we asked whether cardiac pacing function in vivo would be comparable by the two gene transfer modalities. We took advantage of our published work, which employed the same study design with Adv TBX18 instead^50^. Both Adv TBX18 and mRNA TBX18 increased mean heart rates of CAVB rats to a similar degree (Fig. 5g). The mean heart rate of Adv TBX18-treated rats trended higher in the first 4 days post gene delivery. This pattern was similar with control CAVB animals. Both Adv and mRNA GFP-treated CAVB rats exhibited similarly low mean heart rates, but the mean heart rates of Adv GFP-treated rats trended higher early on following injection (Extended Data Fig. 3d). Induction of CAVB leads to acute weight loss^50^, yet no difference was observed in body weights between GFP and TBX18 mRNA-treated CAVB rats during the study period (Extended Data Fig. 3e).

**Fig. 6:**
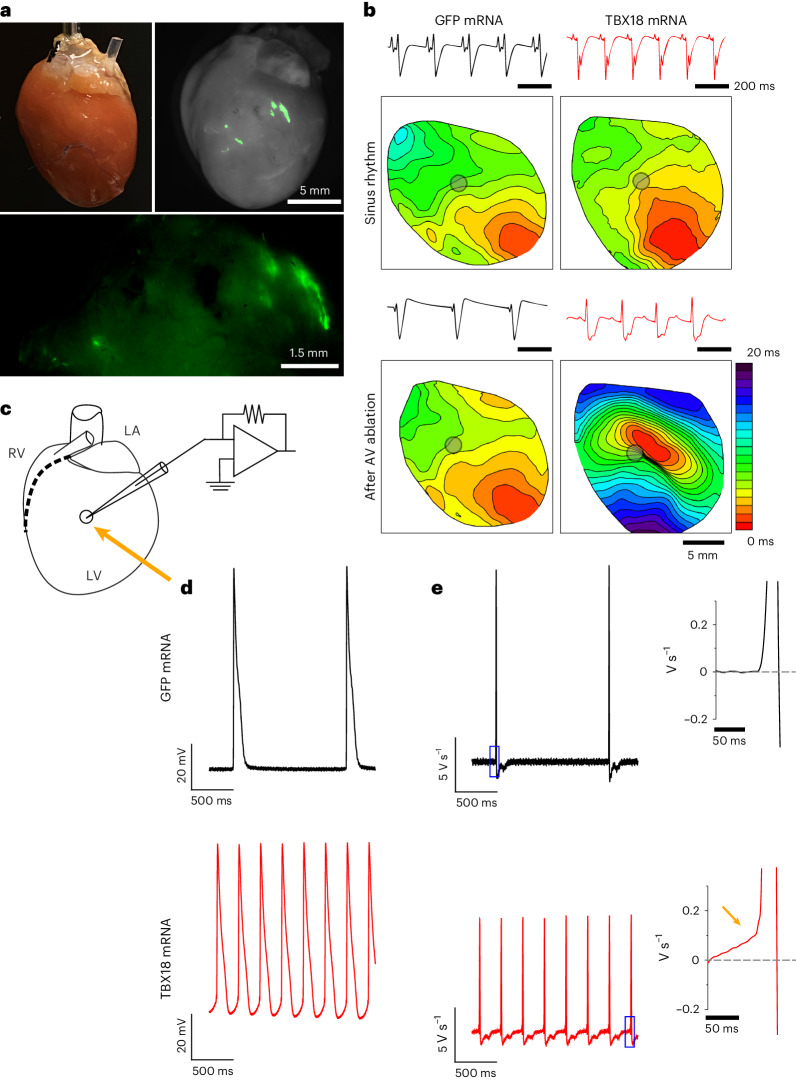
TBX18-induced electrophysiological changes in adult cardiomyocytes in vivo. **a**, Langendorff-perfused rat hearts extracted at 3 days post-mRNA injection. The experiment was repeated independently twice. **b**, Voltage optical mapping contour plots with Di-4-ANEPPS revealed a de novo pacing site near the site of injection (grey circle) in TBX18 mRNA-injected rats following ablation of the AV node (bottom right). No change in electrical propagation was observed under normal sinus rhythm for all rats (top). Simultaneous electrical potential recordings show a change in polarity after AV ablation in TBX18 (above contour) (*n* = 7 rats TBX18, *n* = 3 rats GFP). **c**, An experimental schematic of a sharp electrode recording from Langendorff-perfused rat hearts from the site of injection (arrow) on the LV free wall. LA, left atrium. **d**, Representative traces of action potentials recorded at the site of injection (*n* = 7 rats TBX18, *n* = 3 rats GFP). **e**, Current plots with expanded inset (right) reveal sharp upstroke velocity in GFP, whereas TBX18s show slow phase 4 current.

**Extended Data Fig. 4:**
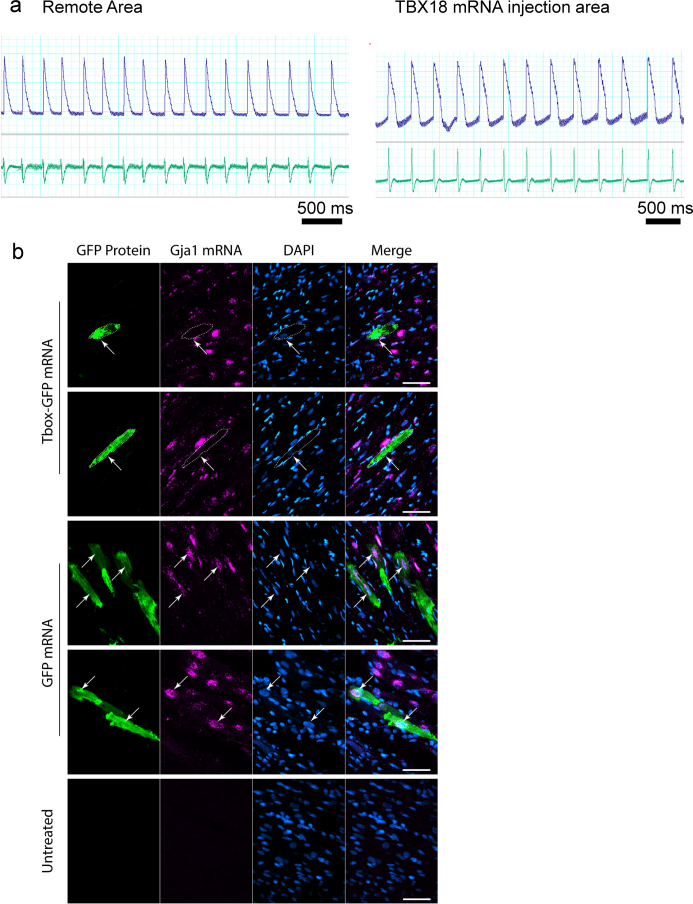
TBX18 mRNA-induced electrophysiological changes in adult cardiomyocytes in vivo. **a**, Representative sharp electrode recordings from TBX18 mRNA-injected rat hearts at a remote site (left) and injection site (right). **b**, RNAscope imaging of rat hearts injected with either TBX18-GFP mRNA (top), GFP mRNA (middle), or unstained (bottom) at 1 day post-injection. Heart sections were co-stained with anti-GFP, Gja1 probe, and DAPI (left to right). White arrows denote nuclei for reference. Scale bar, 50 um. Experiment repeated independently twice.

Furthermore, healthy rat hearts injected with either GFP or TBX18 mRNA at the LV free wall were extracted and retrogradely perfused via Langendorff to study changes in adult myocyte phenotype at 3 days post-injection (Fig. 6a). A loose suture marking the site of injection was confirmed by fluorescence imaging to contain GFP expression (Fig. 6a, inset). Upon loading with Di-4-ANEPPS, voltage optical mapping showed normal electrical propagations originating from the Purkinje fibres near the apex of the free wall (Fig. 6b, top, and Supplementary Videos 6 and 7). Following ablation of the atrioventricular (AV) node, TBX18 mRNA-injected hearts showed relatively slower electrical propagations originating from the injection site (grey circle) with electrical potential recordings showing a change in polarity, while GFP mRNA-injected hearts showed no change from normal sinus rhythm (Fig. 6b, bottom, and Supplementary Videos 8 and 9; *n* = 7 rats TBX18, *n* = 3 rats GFP). Sharp electrode recordings proximal to the injection site on Langendorff-perfused hearts following AV ablation (Fig. 6c) revealed pacemaker-like action potentials in TBX18 mRNA-injected hearts, while GFP mRNA hearts showed relatively slower, normal ventricular action potentials (Fig. 6d). Electrical current plots further confirmed fast upstroke velocities present in the GFP group, while TBX18 showed slow phase 4 depolarization (Fig. 6e; *n* = 7 rats TBX18, *n* = 3 rats GFP). Sharp electrode recordings taken from the remote areas of TBX18-injected hearts showed faster rates with a normal ventricular action potential, while recordings at the injection site were exclusively pacemaker-like (Extended Data Fig. 4a).

Additionally, RNAscope imaging in hearts injected with TBX18-GFP mRNA, as shown in Fig. 3e, showed a distinct absence of *Gja1* (Cx43) transcripts in GFP-expressing cells at 1 day post-injection (Extended Data Fig. 4b), indicating that TBX18-transfected adult myocytes are decoupled from the surrounding myocardium and may explain the slower conduction observed in TBX18-induced propagations.

### TBX18 mRNA creates a biological pacemaker in pigs with CAVB

**Fig. 7:**
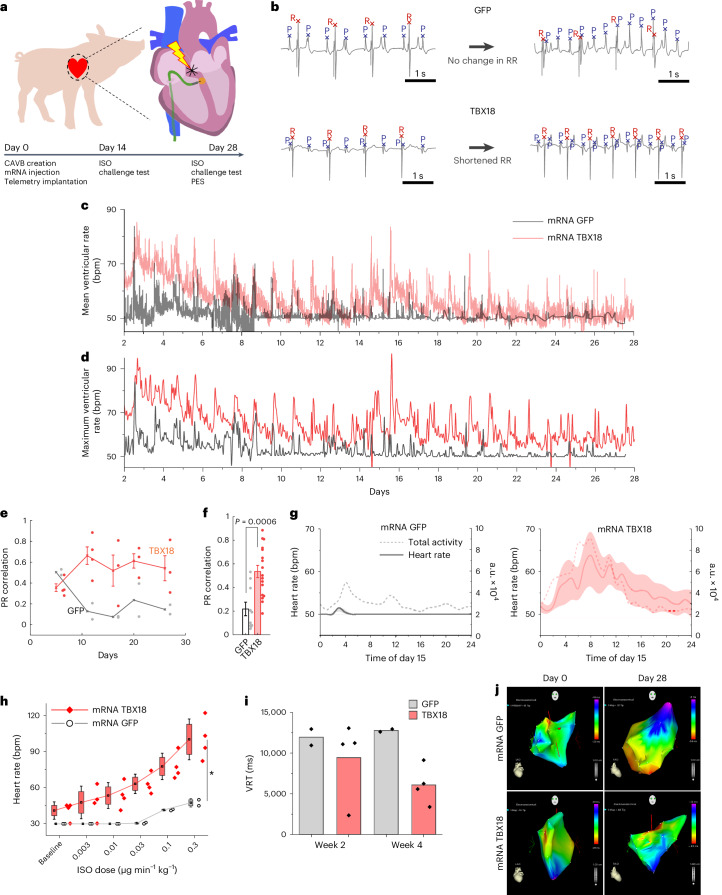
mRNA TBX18 provides heart rate control and chronotropic competence in CAVB pigs. **a**, Large animal study design. **b**, Representative ECG telemetry traces with P and R waves labelled via a machine-learning algorithm. Labelled ECGs reveal correlated shortening of both PP and RR intervals in TBX18 pigs (bottom right). **c**, One-minute averaged ventricular rate, showing circadian cycle and higher heart rates during the 4-week study period in mRNA TBX18 compared with GFP. **d**, The maximum 1-min rate, plotted for each hour, was significantly higher in mRNA TBX18 than control. **e**, The PR correlation for each pig plateaued above the GFP control in TBX18-treated pigs after the first week (*n* = 2 GFP, *n* = 4 TBX18). Mean ± s.e.m. **f**, The overall mean PR correlation was significantly higher in TBX18-treated pigs compared with GFP. *P < 0.05, one-sided two-sample *t*-test, mean ± s.e.m., *n* = 2 GFP, *n* = 4 TBX18 pigs over five timepoints. **g**, The total animal activity (dashed) plotted with heart rate (solid) ± s.d. (shaded area) for each representative pig in mRNA GFP (left) or mRNA TBX18 (right) groups in a 24-h period on day 15. **h**, ISO challenge test given at day (D)28 with increasing doses of β-adrenergic agonist isoprenaline (intravenously infused). Responding heart rates in the TBX18 group were significantly more sensitive, reaching a higher maximal compared with the GFP group (*n* = 2 pigs GFP, *n* = 4 pigs TBX18). *P < 0.05, two-way ANOVA repeated measures Tukey’s test, mean ± s.d. Box, 25th–75th percentiles; whisker, s.d.; centre, mean. **i**, VRT measured from the end of stimulation to the first spontaneous ventricular beat. Mean VRT was lower with mRNA TBX18 at D28 (*n* = 2 pigs GFP, *n* = 4 pigs TBX18). **j**, Representative NOGA images showing intracardiac propagation of antegrade conduction at the mRNA TBX18 injection site (high septal area) to the apex at D28 (right), similar to pre-AV node ablation (left). Red, earliest activation point.

**Extended Data Fig. 5:**
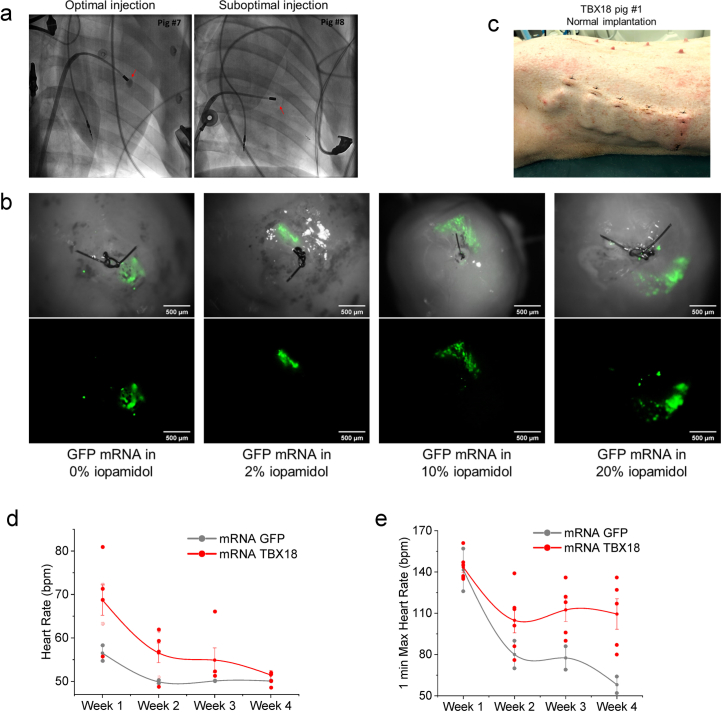
Delivery of mRNA and A83-01 in pigs. **a**, Representative fluoroscopy images showing injection of mRNA and iopamidol solution to the interventricular septum. Red arrows indicate site of injection. **b**, Naked mRNA mixed with various concentrations of iopamidol (contrast agent) injected to the LV apex of rats. Iopamidol (0–20%) had no effect on successful transfection of GFP mRNA. Suture marks site of injection. Experiment repeated independently twice. **c**, Implantation of osmotic pumps to the subcutaneous space of the porcine lower abdomen for systemic delivery of A83-01. **d**, Weekly mean heart rate. **e**, Weekly 1 minute max heart rate. **D, E** n = 2 GFP pigs, n = 5 TBX18 pigs, Mean + /-SEM.

**Extended Data Fig. 6:**
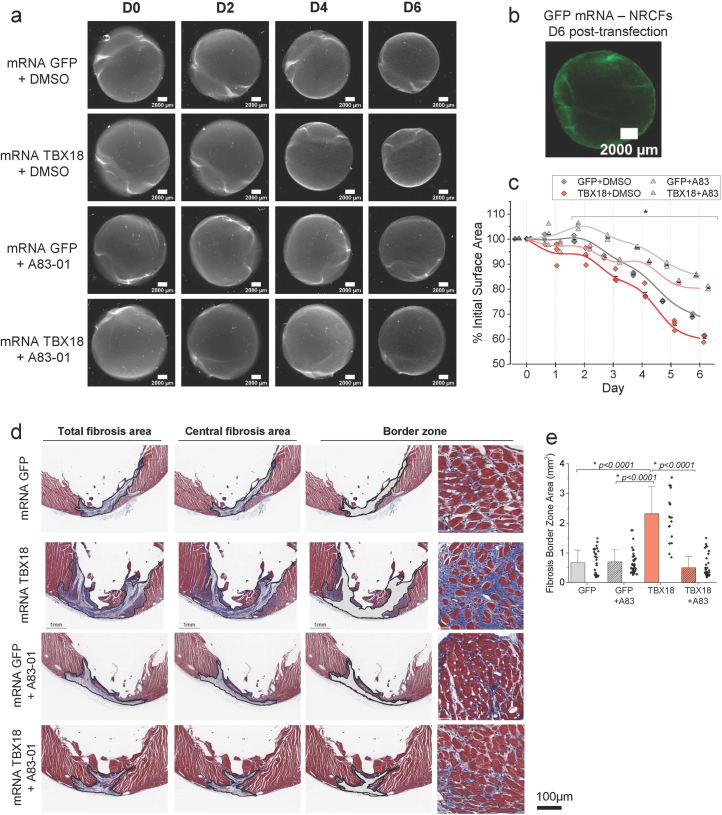
Fibroblast activation and cardiac fibrosis are reduced by treatment with A83-01. **a**, Collagen gel contraction assay revealed TBX18-transfected fibroblasts significantly increased the rate of collagen contraction over the course of 6-days. Scale bar, 2 mm. Experiment repeated independently twice. **b**, Representative fluorescence image of collagen gel loaded with GFP-transfected fibroblasts. Experiment repeated independently twice. **c**, Surface area of collagen gels was measured each day from stereoscope images. Treatment with A83-01 reduced the rate of contraction for all groups (n = 3 gels, *p < 0.05 2-Way ANOVA repeated measures with Bonferroni test. p-value results in Supplementary Table 5). **d**, Representative Masson’s Trichrome stain and measurement of total fibrosis (left), central fibrosis lacking red-stained myocytes (middle), and remaining border zone area (right). Inset shows magnified border zone area with collagen interweaving myocytes. Experiment repeated independently twice. **e**, Fibrosis border zone area, measured from stained heart slices, is significantly higher in TBX18-injected rats, which was significantly reduced with co-treatment of A83 (n = 3 rats, Mean + /-SD, 2-Way ANOVA with Tukey’s Test).

As a key pre-clinical step, we tested mRNA TBX18 as a biological pacemaker in a clinically relevant, porcine model of CAVB^52^. Eight Yorkshire farm pigs were given percutaneous injections of either mRNA TBX18 (*n* = 6) or mRNA GFP (*n* = 2) and monitored for 28 days with ECG telemetry (Fig. 7a). To visually confirm the injection and retention of mRNA to the myocardium, we co-delivered mRNA with the radiopaque agent iopamidol (ISOVUE-M 200), which allowed for real-time fluoroscopic validation of mRNA delivery to the interventricular septum (Extended Data Fig. 5a and Supplementary Video 10). Direct myocardial injection of GFP mRNA mixed with iopamidol to rat hearts revealed no loss of transgene expression, confirming that the contrast agent does not adversely affect mRNA transfection in vivo (Extended Data Fig. 5b). All pigs were treated with the TGF-β inhibitor A83-01 (0.3 mg per kilogram per day) with implanted osmotic pumps over the course of 7 days (Extended Data Fig. 5c), to minimize fibroblast activation and cardiac fibrosis brought on by exogenous TBX18 expression (Extended Data Fig. 6)^53^.

Analysed ECG traces confirmed PR-wave dissociation, representative of complete heart block (Fig. 7b, left). The animals were sustained with a backup pacemaker set at 50 bpm on a demand mode (VVI). Each day at peak hours of heart rate, TBX18-treated pigs were able to respond to increases in sinoatrial rate (PP interval) with significantly shorter RR intervals, while GFP-treated pigs showed no change in ventricular rate, being dependent on the backup pacemaker device (Fig. 7b, right). Over the 4-week study, the minute-by-minute and weekly mean ventricular rate showed a transient increase with regular diurnal oscillations in heart rate for TBX18-treated pigs, compared with the mostly device-paced GFP-treated pigs (Fig. 7c and Extended Data Fig. 5d). This pattern was observed in the 1-min maximal ventricular rate recorded in each hour (Fig. 7d and Extended Data Fig. 5e). Daily increase in the mean heart rate of the TBX18-treated pigs coincided with their once-a-day meal and cage cleaning between 8:00 and 10:00. Outside of these active hours, the pigs remained largely sedentary, signifying that mRNA TBX18-induced biological pacemakers could respond to emotional arousal.

**Extended Data Fig. 7:**
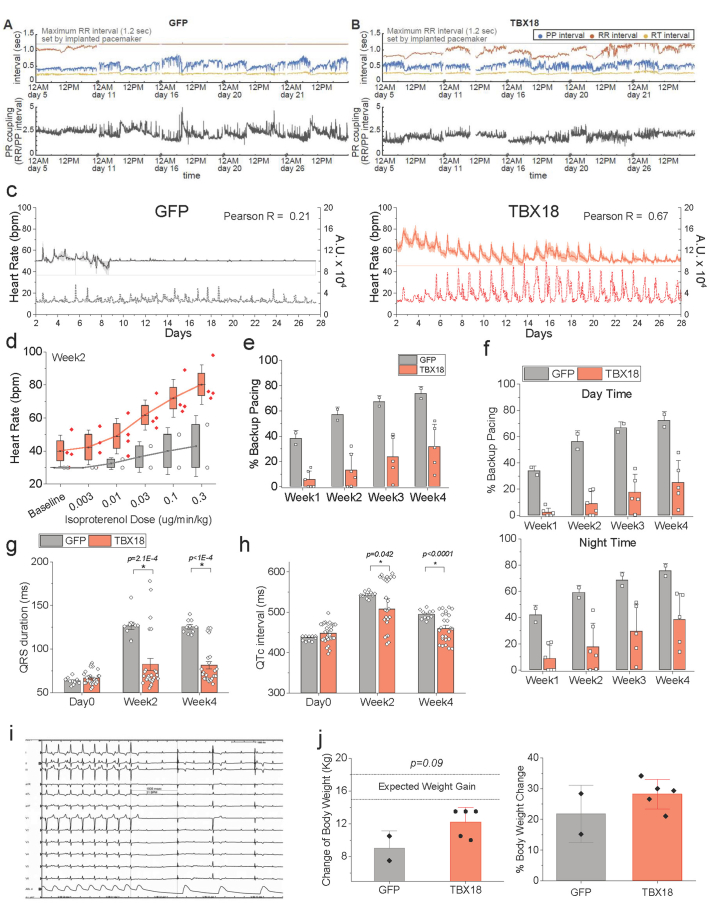
TBX18 mRNA supports higher heart rates and chronotropic competence in CAVB pigs. **a**, **b**, Representative 24-hour plots on select days reveal day-day fluctuations in RR and PP intervals (top) and calculated PR coupling ratio (bottom) from pigs in each group. RR interval of the GFP control pig is at the maximal interval allowed by the back-up pacemaker (1.2 s, 50 bpm, gray line), indicating near complete pacemaker device dependence. **c**, Activity-HR plots for the full 28-day study period. Total activity was highly correlated with heart rate in TBX18 vs. GFP (Pearson coefficient, R = 0.67 vs. R = 0.21 respectively). **d**, Isoproterenol challenge test given at D14 with increasing doses of beta-adrenergic agonist, isoproterenol (i.v. infused, n = 2 GFP pigs, n = 4 TBX18 pigs, Mean +/- SD, Box 25–75 percentile, Whisker SD, Center mean). **e**, Proportion of ventricular beats paced by backup pacemaker. **f**, Pacemaker dependency was lower in day time (top) than night time (bottom). **E,F** n = 2 GFP pigs, n = 5 TBX18 pigs, Mean + /-SD. **g, h**, ECG parameters, QRS duration (**G**) and QT interval (**H**) were significantly shorter in mRNA TBX18 compared to GFP, following AV ablation and mRNA delivery. Mean +/- SEM. (n = 10 GFP, n = 25 TBX18). **G**, *p < 0.05 One-sided Two-sample t test, GFP vs. TBX18 at each time point. **H**, *p < 0.05 One-sided Two-sample t test with Welch Correction, GFP vs. TBX18 at each time point. **i**, Representative ECG trace of VRT measurement. **j**, Increase in body weight for each pig in relation to expected weight gain for healthy pigs (top). TBX18-treated pigs on average had a higher % increase in body weight compared to GFP (bottom). (n = 2 GFP pigs, n = 5 TBX18 pigs, Mean + /-SD, Two-sided Two-sample t Test).

To understand the extent of chronotropic competence, we quantified the degree of correlation between the TBX18-induced induced ventricular pacing (RR interval) with the heart block animals’ sinus rhythm (PP interval) from weeks 1 to 4 post-injection (Fig. 7e). After the animals’ heart rate stabilized past the first week, TBX18-treated pigs exhibited a significant improvement in and consistent PR correlation to the end of the study (Fig. 7e). GFP-treated pigs experienced a significant drop in PR correlation by week 2 with a mean value significantly lower than that of TBX18-treated pigs (0.22 versus 0.53, respectively; Fig. 7f). Plotting PP and RR intervals over select 24-h periods illustrates the ability of mRNA TBX18-induced biological pacing to mimic sinus rhythm fluctuations, while the control animal’s backup pacemaker remained unable to match the animals’ sinus rhythm (Extended Data Fig. 7a,b). We measured the animals’ physical activity with an accelerometer in the implanted telemeter. TBX18-treated pigs exhibited significantly more activity and higher correlation with heart rate, compared with GFP-treated animals in a 24-h period on day 15 (Fig. 7g) and throughout the study (Pearson’s *R* = 0.67 TBX18 versus *R* = 0.21 GFP; Extended Data Fig. 7c). Heart rate response to escalating doses of a β-adrenergic agonist, ISO, in anaesthetized animals indicated that mRNA TBX18-treated pigs were capable of responding to autonomic input with higher heart rate, while control animals’ heart rate was limited to <50% of TBX18’s rate at both days 14 (Extended Data Fig. 7d) and 28 (Fig. 7h). Consequently, dependence on the backup pacemaker device was significantly lower in TBX18-treated animals compared with control (Extended Data Fig. 7e). Greater effect size occurred during the day time than at night, probably due to greater physical and emotional stimuli during the day (Extended Data Fig. 7f). Pacemaker dependency increased for both groups over the course of the study.

The intended site of gene delivery and biological cardiac pacing was the high septal region so as to take advantage of the ventricular conduction pathway. In GFP-treated pigs, the QRS and QTc durations at days 14 and 28 increased substantially from day 0 (before AV node ablation) (Extended Data Fig. 7g,h). In contrast, mRNA TBX18-treated pigs showed significantly shorter QRS and QTc durations compared with control at days 14 and 28, indicating better ventricular synchrony in TBX18-treated animals (Extended Data Fig. 7g,h). Assessing ventricular pacing function, ventricular recovery time (VRT; as a surrogate for sinus node recover time) was significantly shorter in TBX18 compared with control on day 28 (Fig. 7i and Extended Data Fig. 7i). Intracardiac electroanatomical mapping confirmed the earliest activation site (red colour) at day 0, before ablation, was found in the high septum region for all pigs (Fig. 7j). At day 28, GFP-treated pigs showed early activation points arising from the bottom of the right ventricle (RV) at the site of the implanted pacemaker lead (Fig. 7j). In contrast, the earliest activation in TBX18-treated pigs at day 28 originated from the high septum region, the injection site of TBX18 mRNA (Fig. 7j).

As another predictor of clinical outcome, the pigs’ body weights were recorded throughout the study. Both groups were notably below their projected weight gain^54^, given their age and study duration, yet TBX18-treated pigs on average showed higher weight gain than control pigs, closer to their expected growth (Extended Data Fig. 7j). Taken together, the data demonstrate that a single dose of mRNA TBX18 gene transfer creates ventricular pacing for most of the 1-month study duration with evidence for chronotropic competence. The functional efficacy is accompanied with improved indicators of clinical outcome such as greater physical activity and proper growth of the subjects.

### Chronotropic competence of TBX18 mRNA-induced cardiac pacing

**Fig. 8:**
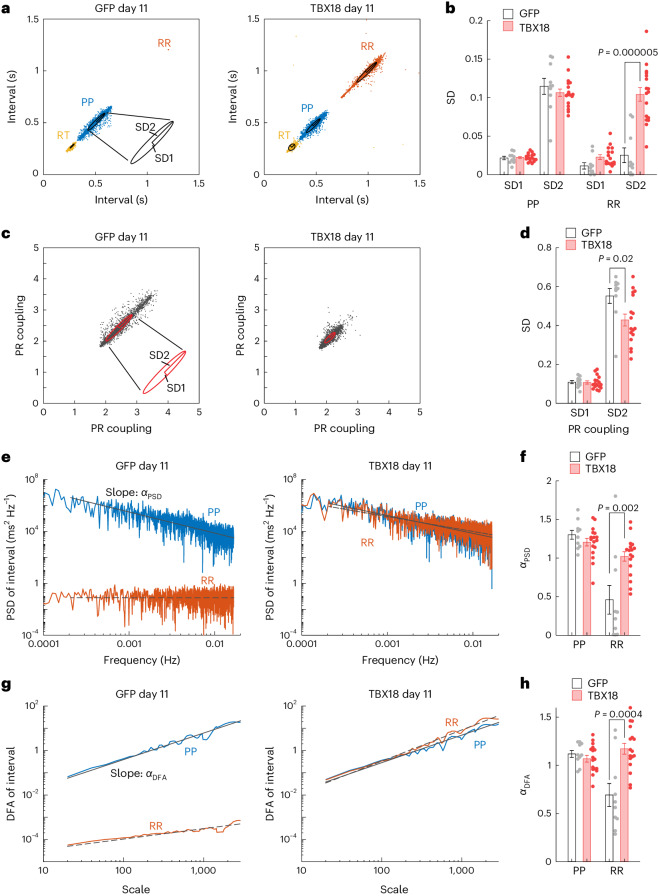
Ventricular rate variability of TBX18 mRNA-paced pigs mimics the variability of their sinus rhythm. **a**, Poincaré plots of PP intervals (blue) and RR intervals (red) at day 11 show minimal RR dispersion in GFP-treated pig (left), compared with TBX18 (right). **b**, Quantification of dispersion measured by oval fitting shows similar SD1 and SD2 values for PP intervals, but significantly lower RR interval SD2 in GFP control. **c**, Poincaré plot at day (D)11 of PR coupling (RR/PP ratio) in GFP and TBX18 pigs. **d**, Oval fitting analysis revealing significantly lower PR dispersion in TBX18-treated pigs. **e**, PSD on D11 of each interval (PP and RR) in the VLF range (<0.04 Hz). **f**, Spectral analysis revealing significantly higher RR interval complexity (*α*_PSD_) in TBX18-treated pigs versus GFP. **g**, DFA of intervals (PP and RR) on D11. **h**, TBX18 pigs had significantly higher RR complexity (*α*_DFA_), compared with GFP. In **b**, **d**, **f** and **h**, mean ± s.e.m. for all pigs on days 5, 11, 16, 20 and 26 combined. *P < 0.05, one-sided two-sample *t*-test. *n* = 2 GFP, *n* = 4 TBX18 pigs over five timepoints.

**Extended Data Fig. 8:**
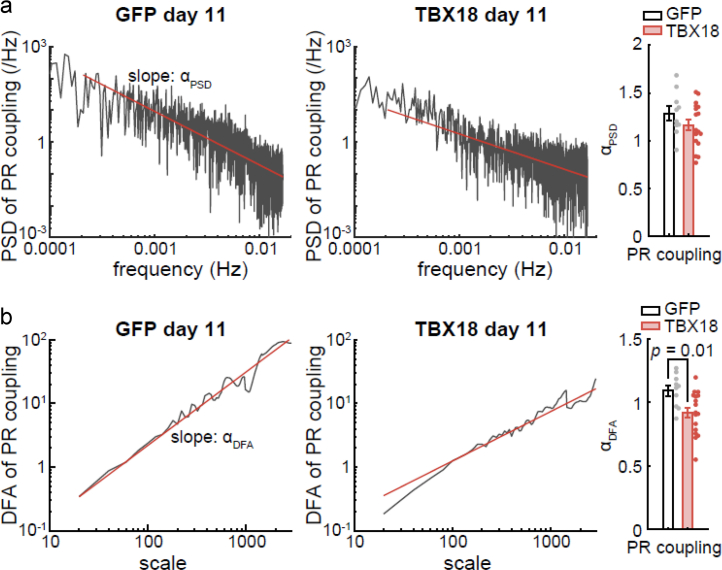
TBX18-treated pigs show lower PR coupling variation than GFP-treated. **a**, Representative power spectral density curves for GFP and TBX18 pigs at D11 (left, middle). Spectral analysis of PR coupling revealed TBX18-treated pigs have a lower average slope (α_PSD_) in the VLF domain, though not significant. Mean +/- SEM. Measured for all pigs (n = 2 GFP or 5 TBX18) at 5 different time points. **b**, Representative detrended fluctuation analysis of PR coupling for all pigs at D11 (left & middle). Fractal complexity measured by the slope (α_DFA_) showed TBX18 pigs had significantly lower PR complexity compared to GFP. Mean +/- SEM. Measured for all pigs (n = 2 GFP or 5 TBX18) at 5 different time points. *p < 0.05 Two-sided Two-sample t test.

We sought to gain insights into safety aspects of mRNA TBX18-paced pigs by examining their heart rate variability. Twenty-four hours of telemetry ECG data were analysed for all pigs at day 11 post-injection, when mean and maximum heart rate stabilized past the first week of surgery. Poincaré plots of PP intervals show similar variation in sinus rhythm between GFP- and TBX18-treated pigs. However, Poincaré plots of RR intervals showed little variability in ventricular rhythm for control animals, due to dependence on the backup pacemaker (Fig. 8a, left). Importantly, the ventricular beat-to-beat variability of mRNA TBX18-paced pigs closely resembled that of their sinus rhythm (Fig. 8a, right). Ellipse fitting analysis confirmed similar SD1 (the instantaneous PP interval variability) and SD2 (the continuous long-term PP interval variability) values in the dispersion of PP intervals between GFP and TBX18, as well as the dispersion of RR intervals along the line of identity (SD2) in TBX18-paced pigs but not in GFP (Fig. 8b). Likewise, PR coupling (RR/PP ratio) in GFP-treated pigs showed large fluctuations, due to the inability of the ventricular rate to adapt to changes in sinoatrial rate, compared with TBX18 pigs (Fig. 8c and Extended Data Fig. 7a,b). Analysis of Poincaré PR coupling showed significantly lower dispersion in TBX18- versus GFP-treated pigs (Fig. 8d). Power spectral density (PSD) analysis further confirmed that PP-interval dispersion is similar between GFP- and TBX18-treated pigs (*α*_PSD_ −1.3 versus −1.2, respectively; Fig. 8e). RR dispersion was significantly higher for TBX18-treated pigs in the very low frequency (VLF; <0.04 Hz) range compared with GFP (*α*_PSD_ −1.02 versus −0.46, respectively; Fig. 8e,f), similar to the PSD of the sinus rate (Fig. 8f). Spectral analysis of PR coupling revealed no significant differences between groups (Extended Data Fig. 8a). Detrended fluctuation analysis (DFA), which can account for potential dependence between time series data points, also indicated that mRNA TBX18 pigs showed nearly overlapping PP and RR complexity (Fig. 8g). Fractal complexity, measured by the slope of DFA, was similar between the PP and RR intervals of mRNA TBX18-paced pigs and significantly more complex than the RR intervals of GFP-treated pigs (*α*_DFA_ 1.17 versus 0.69, respectively; Fig. 8h). Overlap in PP and RR interval complexity resulted in the significantly lower complexity in PR coupling of TBX18 compared with control GFP pigs (Extended Data Fig. 8b).

**Extended Data Fig. 9:**
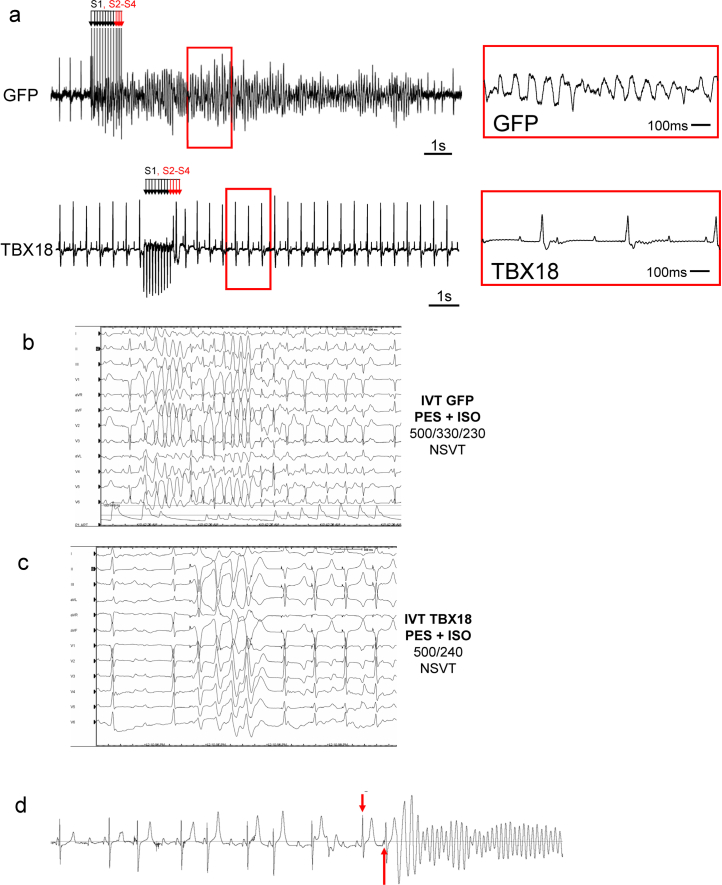
PES results for all subjects. Induction or non-induction of arrhythmia with PES +/- Isoproterenol, β-adrenergic stimulation. **a**, Representative surface ECG results of GFP and TBX18-treated rats (n = 6 rats TBX18, n = 5 rats GFP). Arrows indicate when electrical stimulation was applied. No rats showed sustained tachyarrhythmias (>30 s). Both treatments showed similar prevalence of inducible non-sustained arrhythmias. **b, c** Representative surface ECG traces of GFP and TBX18-treated pigs with inducible non-sustained ventricular tachycardia (NSVT). Akin to rats, prevalence of arrhythmia was similar between treatments in pigs. **d**, Telemetry ECG trace of spontaneous ventricular fibrillation in a pig treated with TBX18 mRNA.

**Extended Data Fig. 10:**
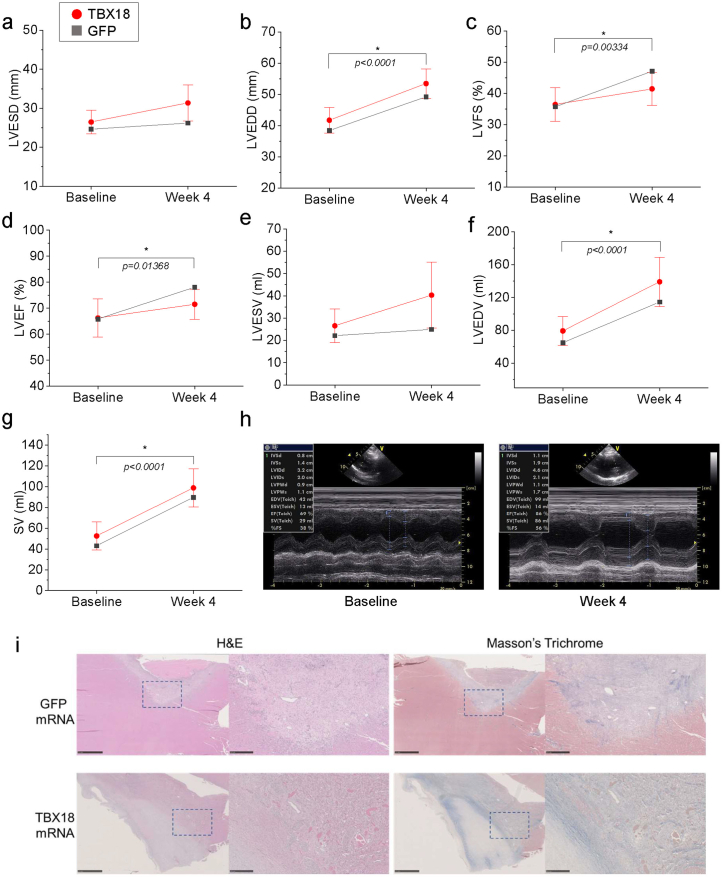
Echocardiography of LV chamber dimension and functional changes before and after the study. TBX18 (red), GFP (gray). Measurements made with M-mode. **a**, Left ventricular end systolic diameter. **b**, Left ventricular end diastolic diameter. **c**, Left ventricular fractional shortening. **d**, Left ventricular ejection fraction. **e**, Left ventricular end systolic volume. **f**, Left ventricular end diastolic volume. **g**, Stroke volume. **h**, Representative M-mode echo images at baseline and week4. All pigs showed LV chamber dilation, most notably by diastolic diameter and volume increase. Enlarged LV chambers resulted in significantly higher stroke volume by the end of the study. No statistical differences were measured between treatment groups. Mean +/- SD. (n = 2 GFP, 5 TBX18, 2-way ANOVA) **i**, Histological staining of pig heart tissues collected near the site of AV ablation show similar amounts of necrosis and fibrosis in GFP-(top) and TBX18-(bottom) mRNA injected pigs. Experiment repeated independently twice. Scale bar, 2.5 mm.

Imbalance in autonomic regulation can provide both the trigger and substrate for ventricular arrhythmias^55^. We examined whether the heightened β-adrenergic responsiveness of mRNA TBX18-induced ventricular pacing changed arrhythmogenic propensity in both small and large animal models. All animals were examined in the context of chronic and complete heart block, increasing their susceptibility to induced arrhythmias by programmed electrical stimulation (PES). Sustained (>30 s) ventricular tachycardias were not observed in any small animals. PES induced non-sustained ventricular tachycardias in four of five GFP-injected rats and in three of six mRNA TBX18-injected rats (Extended Data Fig. 9a). In pigs, no difference in the rate of PES-induced tachyarrhythmia was observed between GFP- and TBX18-treated pigs with instances of non-sustained ventricular tachycardia occurring only in the presence of ISO infusion combined with PES (Extended Data Fig. 9b,c). We observed one incidence of unexpected death in a pig treated with TBX18 mRNA, experiencing spontaneous sustained ventricular fibrillation, the cause of which could not be determined (Extended Data Fig. 9d). During this 1-month study, differences in ventricular remodelling due to bradycardia were not observed between GFP- and TBX18-treated pigs. Both groups of animals exhibited significantly increased LV end diastolic volume and stroke volume from the start to the end of the study (Extended Data Fig. 10a–h). Similar degrees of necrosis and fibrosis were observed at the ablation site of GFP and TBX18 pigs (Extended Data Fig. 10i).

## Discussion

This study demonstrates use of an mRNA-based gene therapy to generate cardiac biological pacemakers in small and large animal models of complete AV block. A single dose of synthetic TBX18 mRNA to the myocardium transiently increased the heart rates of rats and pigs in severe bradycardia over the course of 2 or 4 weeks, respectively. TBX18 mRNA-treated pigs demonstrated significantly high PR correlation (Fig. 7e), chronotropic competence (Fig. 7g) and reduced dependence on a backup pacemaker (Extended Data Fig. 7e). The benefits of mRNA over Adv vectors for TBX18 delivery shown here include little to no off-target biodistribution and lower immune/inflammatory response. Together with minimally invasive percutaneous gene delivery and real-time validation of gene delivery under fluoroscopy, our study provides grounds for mRNA TBX18 as a safer gene-based biological pacemaker for clinical translation.

Our data demonstrate that synthetic mRNA delivery is capable of achieving functional gene transfer to cardiomyocytes with high efficiency (>75%; Fig. 1e) and for up to 7 days (Fig. 1c). Protein translation was rapid and robust, detectable within 4 h of transfection (Fig. 1a). In contrast, Adv transgene expression lingered for a considerably longer period of time in myocytes, lasting more than 2 weeks (Fig. 1b). It was unexpected that in vitro transfection of mRNA was more effective into cardiomyocytes than into non-myocytes (Fig. 1e). It may be an inherent property of the mRNA or the transfection reagent used for in vitro experiments, or both. In line with a previous study^31^, our data demonstrate that direct myocardial gene transfer of mRNA in vivo does not require transfection reagent (Fig. 3a). Synthetic mRNA was dissolved in sterile, RNase-free saline for all in vivo experiments in this study. This formulation is expected to simplify the product development pipeline for clinical translation of synthetic TBX18 mRNA-based gene therapy^56^.

Continuous, 24/7 ECG telemetry recordings from ambulatory CAVB rats revealed that ventricular pacing induced by TBX18 mRNA gene transfer displayed at least two distinct QRS complex morphologies (Fig. 5b and Extended Data Fig. 3b). One QRS complex morphology was faster in rate and negative in polarity on lead II, indicating retrograde conduction (Fig. 5b, arrows). The other was slow in rate and positive in polarity (Fig. 5b, circles), resembling the slow junctional rhythm from the control, GFP mRNA-injected rats. The general myocardium, with its robust inward rectifier K^+^ current (*I*_K1_) density^57,58^, is strongly hyperpolarizing and not conducive to spontaneous electrical activity. The LV apex as the site of TBX18 mRNA delivery was by design, so as to readily identify de novo pacing as negative QRS complexes due to retrograde pacing. The de novo pacing site in TBX18 mRNA-treated rats was manifested as the faster peak in a bimodal distribution of all heartbeats (Fig. 5d). It is plausible that the faster retrograde pacing and slow junctional rhythm may have been complementary rather than competing in TBX18 mRNA-injected animals.

To overcome source–sink mismatch^59,60^, between de novo TBX18 pacing site and the ventricular myocardium, and achieve ventricular synchrony, we delivered the transgene to the high His bundle region with an intramyocardial injection catheter in a large animal model, providing better insight into the clinical effectiveness of TBX18 mRNA to treat bradycardia^15,16,52^. Given the requirement of focal gene transfer for cardiac pacing and labile nature of naked mRNA, it is critical to validate successful retention of mRNA upon myocardial injection. Here, we report that radiographic contrast agent iopamidol (ISOVUE-M 200) did not interfere with the transfection of naked modified mRNA when co-injected with GFP mRNA to the LV myocardium of rats (Extended Data Fig. 5b). This method was directly scaled up for pigs, allowing for real-time visualization of injected TBX18 mRNA to the His bundle region (Extended Data Fig. 5a). Co-delivery of iopamidol and other modified mRNAs may offer a vital tool for successful percutaneous injection in other therapeutic applications.

Our previous work demonstrated that TBX18-induced pacemaker cells recapitulate hallmark features of SAN pacemaker cells and can serve as a surrogate tissue model for the SAN^13,14,61–63^. Tbx18 is an embryonic transcription factor^64^ with a short half-life^65^, implicating the transient nature of its protein expression. Noting that somatic cell reprogramming can be achieved within days of transgene expression^43,66,67^, we reasoned transient expression of TBX18 mRNA could be sufficient for inducing automaticity from ventricular cardiomyocytes. Our data demonstrate that TBX18 mRNA is capable of inducing notable electrophysiological changes in ventricular myocytes, with de novo automaticity and weak cell–cell coupling in less than a week after gene transfer (Fig. 2a–c,i). Three lines of evidence support the notion that the brief expression window of synthetic mRNA suffices to provide a transient biological pacemaker. First, the induced automaticity is accompanied by significant increases in *Hcn4* transcripts within the first 3 days of transfection and increased levels of pacemaker-related genes after 2 weeks (Fig. 2d and Extended Data Fig. 1i). Second, cardiomyocytes isolated from *Hcn4*^(GFP/+)^ transgenic mice indicate that TBX18 mRNA transfection induced a significant rise in the number of de novo Hcn4^+^ myocytes, and the Hcn4^+^ proportion was comparable to Adv TBX18 at day12 (Fig. 2e,f). NMVMs exhibited a baseline of 1.5% GFP^+^ myocytes for both mRNA and Adv controls, probably representing ventricular conduction cells and the immature nature of the cells (Fig. 2f). From this baseline, we approximate a 1.5% conversion efficacy, achieving 3% GFP-expressing cells after TBX18 transfer with either vector. Third, a single dose of TBX18 mRNA provided functional ventricular pacing in both rats and pigs with CAVB (Figs. 5b,c, 6b and 7b,c).

Work by us and others on gene-based cardiac biological pacemakers has relied on the use of recombinant Adv vectors to deliver the transgene^14,52,68–73^. Although effective, the viral vectors have been reported to elicit strong innate and adaptive immune responses, increasing their toxicity and limiting safety profile^74,75^. Our data showed that recruitment of infiltrating leukocytes and elevated inflammatory cytokine levels with Adv gene delivery were all significantly reduced with mRNA gene transfer (Fig. 4c,d and Supplementary Fig. 2b–d). On the other hand, adeno-associated virus vectors have been validated as a relatively safe and effective gene therapy modality with regulatory approvals in the United States for human application. Particularly, systemic delivery of adeno-associated viruses with cardiac tropism can transduce large regions of myocardium. This is suitable for applications that require organ-level and long-term transgene expression, but not for direct reprogramming strategies that require transient and focal expression of transgenes. Recent report of a cardiomyocyte-specific modified mRNA translation system further strengthens specificity of mRNA-based gene therapies^76^. Taken together, our data demonstrate that focal and transient expression, minimal immunogenicity (Fig. 4c,d) and undetectable off-target expression (Fig. 4a,b) make synthetic mRNA-based gene transfer an ideal modality for biological pacemaker applications.

For clinical translation, the criteria for therapeutic efficacy may vary according to disease indications. For example, a temporary biological pacemaker would need to be fully reliable if used as a bridge-to-device therapy. For long-term pacing with a backup device, >60% biological pacing may suffice to improve the pacemaker-induced cardiomyopathy that is associated with chronic RV pacing^16^. A potential indication for biological pacing is for patients with artificial pacemakers whose device becomes infected^6,11^. Biological pacemakers could provide hardware-free, bridge-to-device alternatives during infection treatment until implantation of a definitive device. The considerably decreased immunogenic profile of modified mRNA would reduce potential complications of employing viral vector-based gene transfer for patients with infection as well as paediatric patients with heart block. Recent advances in bioresorbable electronics have opened the door for temporary cardiac pacing devices^77^. However, practicality of this technology is limited by inductive charging, requiring a strong magnetic field. Our findings demonstrate the feasibility of mRNA-based biological pacing towards clinical translation by demonstrating transient efficacy in a non-viral vector, particularly for patients for whom viral gene therapy may not be suitable.

### Durability of biological pacing

The 24-h mean heart rates of TBX18 mRNA gene therapy appear to wane over the course of the 4-week study (Fig. 7c and Extended Data Fig. 5d), similar to ventricular pacing achieved by adenovirus-mediated TBX18 gene transfer in previous studies^15,16^. The gradual loss of function may be due to potential loss and/or reversion of partial or fully reprogrammed pacemaker cells. However, the maximum heart rates (maximum 1-min heart rate from each hour, 24 datapoints per day in Fig. 7d, and weekly maximum heart rates in Extended Data Fig. 5e) appear to stabilize after the first week. Furthermore, the diurnal changes in the heart rate (Fig. 7g, solid line) are sustained throughout the 4-week study period (Fig. 7c,d). To better understand durable function, mechanistic studies are required, including epigenetic stability of reprogrammed cells, minimal duration and/or dosing of transgene expression as well as minimum number of reprogrammed cells required to overcome source–sink mismatch^78^.

### Mechanism of reprogramming

We have used the term reprogramming and conversion to pacemaker cell interchangeably, noting their functional aspects such as *Hcn4* expression and de novo pacemaker phenotype. From a molecular perspective, stable reprogramming implies permanent conversion of epigenetic landscape from source to target cell, which we do not yet understand. Here, we have shown TBX18-transfected cardiomyocytes gain part of the gene identity of pacemaker cells (Fig. 2d and Extended Data Fig. 1i; Hcn4 and Tbx3). Nonetheless, thorough epigenetic studies are needed to determine the partial and/or fully reprogrammed pacemaker state of these cells. Investigation of dynamic changes to epigenetic and transcriptional landscapes of TBX18-reprogrammed pacemaker cells together with lineage tracing will facilitate defining the durability of TBX18-induced cardiac pacing at single-cell resolution.

### Extrinsic factors that may elicit ectopic electrical beats

We cannot rule out yet-to-be-determined factors that are extrinsic to the biological pacemaker impacting its function over time. For instance, spontaneous activity induced by cytotoxicity has not been ruled out. General innate immune response to synthetic mRNA may confound interpretation of the functional data that TBX18 mRNA gene transfer led to de novo pacing at the site of gene transfer in the rodents (Fig. 6) and in the large animal model (Fig. 7). Innate immune activity of TBX18 versus GFP mRNA was not examined here, which may impact TBX18-induced automaticity mechanism to generate pacing. As a prelude to clinical translation, dedicated studies will be required to understand innate immune response to TBX18 mRNA-mediated gene therapy with mRNA formulations that reflect US Food and Drug Administration guidelines on mRNA gene delivery methods.

### Clinical translatability

In future efficacy studies, alternative large animal models such as sheep should be considered^79^, which are more conducive to >6 months long-term follow-up periods and less sedentary than the porcine model employed here. Furthermore, the efficacy of this mRNA gene therapy could be considerably enhanced by conjugating the naked mRNA with lipid nanoparticles or antagomiRs shown to extend TBX18 expression^80^. The coronavirus disease 2019 mRNA vaccines have proven the effectiveness of lipid nanoparticles in enhancing mRNA gene transfer at substantially lower doses, as well as their safety and efficacy for re-dosing. Future work is necessary to determine if higher pacing efficiency can be extended through other strategies, including but not limited to mRNA dosing, increasing transfection efficacy or re-dosing at multiple timepoints. Use of transfection reagents may aid in improving the transfection efficiency in vivo, which in turn may increase pacing efficacy and duration. However, these carriers may increase the risk of off-target transfection, which will need to be weighed against the added benefit.

## Methods

All experiments involving animals were performed in accordance with approved protocols from the Institutional Animal Care and Use Committee (IACUC) and the Division of Animal Resources (DAR) of Emory University School of Medicine.

### IVT mRNA synthesis

Plasmids for IVT mRNA synthesis were obtained from GeneArt Synthesis and Services (Thermo). Gene sequences for enhanced GFP, fLuc and TBX18 were codon optimized using the GeneArt codon optimization tool and inserted into a pMA7 backbone containing a T7 promoter and a NotI restriction site. IVT mRNA was then produced by T7-polymerase-based in vitro run-off transcription from the linearized DNA template using a HiScribe T7 kit (New England Biolabs). Nucleotides used were ATP, CTP, GTP and *N*^1^-methylpseudouridine-5′-triphosphate (Trilink). Upon in vitro transcription of the mRNA, the template plasmid DNA was degraded by DNase I digestion (Alevron), and the mRNA was purified by lithium chloride precipitation (Thermo). The IVT mRNA was enzymatically capped and tailed using enzymes from the in vitro transcription with Enzymatic Capping & Tailing Kit (Aldevron). IVT mRNA was then purified before and after treatment with Antarctic Phosphatase (New England Biolabs). Full-length transcription of each batch of IVT mRNA was confirmed by running on a 1.8% agarose gel to ensure a single band is present. To confirm robust protein translation, each batch of IVT mRNA was transfected into HeLa cells using Lipofectamine 2000 (Thermo). Protein expression was assessed 24 h later by a luciferase assay (fLuc), flow cytometry (eGFP) or immunofluorescence against a FLAG epitope tag (TBX18) (Sigma Aldrich; #F3165; 1:1,250).

### Myocyte isolation and transfection

NRVMs and NMVMs were isolated from 2- to 3-day-old neonatal rat pups and cultured as a monolayer as described previously^81,82^. The left and right ventricles were isolated from the rest of the heart by cutting the bottom half of the heart. For all in vitro experiments, NRVMs and NMVMs were plated at a density of 210,000 cells cm^−^^2^. All NRVM culture and transfections were performed in a routine NRVM culture medium based on M199 with the following components: 10 mM HEPES, 0.1 mM non-essential amino acids, 3.5 mg ml^−1^ glucose, 2 mM GlutaMAX (ThermoFisher Scientific), 4 μg ml^−1^ vitamin B12, 100 U ml^−1^ penicillin and heat-inactivated FBS at 10% (first 2 days of culture) or 2% (after 2 days of culture) final concentration. All myocyte cultures were transfected with ViromerRed (OriGene Technologies) according to the manufacturer’s protocol. The dose of mRNA delivered was standardized to the number of cells cultured (ng per 1,000 cells). Unless otherwise indicated, NRVMs and NMVMs were incubated with the complexed mRNA–Viromer solution overnight (<12 h). The IVT mRNA was washed out and replaced with fresh medium after incubation. All myocytes were plated and cultured for at least 24 h before transfection. For high-throughput assessment of automaticity, NRVMs were seeded and transfected on 48-well CytoView MEA plates (Axion Biosystems). Extracellular potentials were recorded for 30 min at each timepoint under a live cell environmental chamber, controlling heat and carbon dioxide. The day before transfection (−24 h) one recording was done to determine viable wells with detectable, active beating. Active wells were defined as beating greater than 5 bpm. Inactive wells were discarded and not transfected.

### In vivo rodent mRNA delivery

All in vivo experiments were performed in accordance with approved protocols from the IACUC and the DAR of Emory University School of Medicine. For all in vivo experiments, 300 µg of IVT mRNA was dissolved in 100 μl of RNase-free, sterile saline (cat. no. 341005, Bioo Scientific) and delivered to the apex of the left ventricle without the use of transfection reagents. Equivolume of Adv vectors (Ad-CMV-GFP cat. no. 1060 and Ad-CMV-Luciferase cat. no. 1000, Vector Biolabs) were injected at a dose of 0.5 × 10^9^ PFU. Before surgery, animals were anaesthetized with 5% isoflurane for 6 min and placed on a mechanical ventilator after intubation. Anaesthesia was maintained with 2% isoflurane during the surgery. Body temperature was maintained at 37 °C during surgery using a heated water bath. Meloxicam (5 mg kg^−1^) and Buprenorphine SR (1 mg kg^−1^) were delivered subcutaneously for analgesia. Normal Sprague Dawley rats of 250–350 g body weight were subjected to partial right thoracotomy to create an ambulatory model of CAVB as we have previously reported^40^. Briefly, the AV node region, with its characteristic fat pad, was exposed by ligating and retracting the right atrial appendage. Monopolar electrosurgical current was delivered subepicardially to the AV nodal region via a sharp needle. Upon creating CAVB, animals were monitored for 1 week with surface ECG to confirm stable 3° block. A dual-biopotential telemetry device was implanted to record 24/7 ambulatory ECG from each animal for 2 weeks post-gene delivery.

### In vivo porcine mRNA delivery

All animal surgical procedures and care were approved by IACUC of Emory University School of Medicine. Four-month-old female domestic Yorkshire crossbred swine (40–50 kg body weight) were enroled in this study. Lyophilized IVT modified mRNA (3 mg) was mixed with 0.5 ml of RNase-free saline and 0.5 ml of 41% iopamidol injection (ISOVUE-200, Bracco Diagnostics). NOGA Myostar injection catheter (Johnson & Johnson) needle length was set to 5 mm with the catheter tip banded 90°. One millilitre of prepared mRNA–iopamidol solution was delivered at the upper interventricular septum from the RV side with the NOGA Myostar injection catheter. Each injection of 300 µl was slowly ejected over the course of 1 min, for a total of three injections in each animal. After each injection, the needle remained in the tissue for 30 s to prevent regurgitation of modified mRNA from the injection site. One-hundred microlitres of RNase-free saline was flushed through the NOGA Myostar injection catheter after the third injection. Optimal injection was confirmed with fluoroscopic imaging, observing a focal radio-opaque spot at the injection site for 5 min (Supplementary Video 10). An additional injection of 300 µl modified mRNA was provided if a suboptimal injection was observed with fluoroscopy. Twenty-six milligrams of TGF-β inhibitor, A83-01, was dissolved in 0.52 ml of dimethyl sulfoxide and diluted in 4.68 ml of corn oil. Five millilitres of prepared A83-01 solution was injected subcutaneously. One-hundred milligrams of A83-01 was dissolved in 1 ml of dimethyl sulfoxide and diluted with 9 ml of corn oil. Ten millilitres of diluted A83-01 was loaded in five osmotic pumps (Alzet 2ML1), designed for continuous release for 1 week, and implanted subcutaneously on the right side of the thoracic cage (Extended Data Fig. 5c).

### Reporting summary

Further information on research design is available in the Nature Portfolio Reporting Summary linked to this article.

## Supplementary information

Supplementary Information Supplementary methods, figures, tables, references and video captions.

Reporting Summary

Supplementary Video 1 Adv versus IVT GFP time lapse.

Supplementary Video 2 Optical mapping of voltage change on fLuc-transfected monolayers paced at 1–3 Hz.

Supplementary Video 3 Optical mapping of voltage change on TBX18-transfected monolayers paced at 1–3 Hz.

Supplementary Video 4 Near-IR tagged IVT mRNA myocardial injection 1.

Supplementary Video 5 Near-IR tagged IVT mRNA myocardial injection 2.

Supplementary Video 6 Optical mapping of GFP mRNA-injected rat heart under sinus rhythm.

Supplementary Video 7 Optical mapping of GFP mRNA-injected rat heart after AV node ablation.

Supplementary Video 8 Optical mapping of TBX18 mRNA-injected rat heart under sinus rhythm.

Supplementary Video 9 Optical mapping of TBX18 mRNA-injected rat heart after AV node ablation.

Supplementary Video 10 Fluoroscopic video of percutaneous mRNA injection with iopamidol to the pig heart.

## Source data

Source Data Fig. 2 Unprocessed western blots for Fig. 2i.

Source Data Extended Data Fig. 1 Unprocessed western blots for Extended Data Fig. 1b.

## Data Availability

The main data supporting the results of this study are available within the paper and its Supplementary Information. All other source data, including the analysed rat and pig telemetry datasets related to Figs. 5, 7 and 8, which are too large to be shared publicly, are available for research purposes from the corresponding authors on reasonable request. Source data are provided with this paper.

## Code availability

Code used for the analysis of in vitro optical mapping, related to Fig. 2g, was based off previously published work (10.1161/CIRCULATIONAHA.119.039711). Analyses of optical mapping of ex vivo rat hearts, related to Fig. 6b, were done with a custom-built software program developed in Interactive Data Language, publicly available via GitHub at https://github.com/arvinsoepriatna/AP_Analysis_Routines_Cardiotoxicity_Microtissues. All codes used for the data analyses are available from the corresponding authors on request.
