# Supplementary material for: Transient pacing in pigs with complete heart block via myocardial injection of mRNA coding for the T-box transcription factor 18
Source: Nat Biomed Eng. 2024 May 2;8(9):1124–41. doi: 10.1038/s41551-024-01211-9 (PMC11410671; doi:10.1038/s41551-024-01211-9)
Supplement: Supplementary file 1 — Supplementary methods, figures, tables, references and video captions. [file 41551_2024_1211_MOESM1_ESM.pdf]

nature biomedical engineering

Article https://doi.org/10.1038/s41551-024-01211-9

Transient pacing in pigs with complete heart block via myocardial injection of mRNA coding for the T-box transcription factor 18

In the format provided by the authors and unedited

Contents

Detailed Methods

Supplementary References

Uncropped Gel Images related to Fig. 2i and Extended Data Fig. 1b

Supplementary Figures

Supplementary Fig. 1: Flow cytometry gating strategy in NRVMs

Supplementary Fig. 2: Heart and global biodistribution of IVT mRNA expression *in vivo*.

Supplementary Tables

Supplementary Table 1: 2-way ANOVA Means Comparisons related to Fig. 2f. IVT mRNA TBX18 vs. fLuc.

Supplementary Table 2: 2-way ANOVA Means Comparisons related to Fig. 2f. Adv TBX18 vs. Adv mCherry.

Supplementary Table 3: 2-way ANOVA Means Comparisons related to Extended Data Fig. 1g. Adv TBX18 vs. Adv mCherry.

Supplementary Table 4: 2-way ANOVA Means Comparisons related to Extended Data Fig. 1g. IVT mRNA TBX18 vs. fLuc.

Supplementary Table 5: 2-way ANOVA Means Comparisons related to Extended Data Fig. 6c. IVT mRNA TBX18 vs. GFP +/-

A83-01 Supplementary Videos

Supplementary Video 1: Adv vs IVT GFP time-lapse

Supplementary Video 2: Optical mapping of voltage change on fLuc-transfected monolayers paced at 1-3Hz.

Supplementary Video 3: Optical mapping of voltage change on TBX18-transfected monolayers paced at 1-3Hz.

Supplementary Video 4: Near infrared tagged IVT mRNA myocardial injection #1

Supplementary Video 5: Near infrared tagged IVT mRNA myocardial injection #2

Supplementary Video 6: Optical mapping of GFP mRNA injected rat heart under sinus rhythm.

Supplementary Video 7: Optical mapping of GFP mRNA injected rat heart after AV node ablation.

Supplementary Video 8: Optical mapping of TBX18 mRNA injected rat heart under sinus rhythm.

Supplementary Video 9: Optical mapping of TBX18 mRNA injected rat heart after AV node ablation.

Supplementary Video 10: Fluoroscopic video of percutaneous mRNA injection with iopamidol to the pig heart.

Detailed Methods:

IVT mRNA Fluorescent Labeling

IVT mRNA was fluorescently labeled using multiply labeled tetravalent RNA imaging probes (MTRIPs), which bind, via nucleic acid hybridization, to the 3' untranslated region (UTR) of the mRNA, as previously described.[1] Briefly, 2'-O-methyl RNA-DNA chimeric oligonucleotides 15-17 nucleotides in length containing three or four amino-modified thymidines, a short poly(T) linker, and a 5' biotin were obtained (Biosearch Technologies). Three oligonucleotides complementary to sequences in the 3' UTR of the mRNA were used for annealing. The oligonucleotides were labeled with Dylight 680-NHS esters (Pierce) using manufacturer's protocols and excess dye was removed using 3kDa centrifugal filters (Millipore). MTRIPs were assembled by incubating a Neutravidin core with each fluorescently labeled oligonucleotide at a 1:5 molar ratio for 1 hour at room temperature. Excess oligonucleotides were removed using 30kDa filters. For mRNA labeling with MTRIPs, mRNA, was first heated at 75°C for 10 min to remove secondary structure and immediately placed on ice for 2 min. MTRIPs were added to the mRNA and incubated at 37°C for 2 hours. The mRNA-to-MTRIP ratio was optimized to 3:1, which ensured efficient mRNA labeling while avoiding the need for a final purification step by filtration. For *in vivo* experiments requiring the visualization of fluorescently labeled mRNA, the above protocol was performed immediately prior to injection.

Flow cytometry

Flow cytometric analysis was conducted on NRVMs transfected with IVT GFP mRNA as described above. Briefly, NRVM monolayers were dissociated to a single cell suspension using 0.05% Trypsin-0.53mM EDTA. Suspended cells were stained using LIVE/DEAD™ Fixable Blue Dye (Invitrogen) according to the manufacturer's instructions. Cells were then fixed with 4% paraformaldehyde and stored in FACS Buffer (1x PBS + 1% Heat Inactivated FBS) at 4°C until staining was performed at a later timepoint (up to 3 days later). Immediately prior to staining, cells were incubated in Perm/Wash Buffer (BD, 554723) for 20 min at 4°C. Cells were washed with FACS buffer and incubated in a solution containing the primary antibodies sarcomeric alpha-actinin (Sigma-Aldrich; #A7811; 1:4,500) and GFP-Booster (Chromotek, #gba488-100, 1:800) for 30 min at 4°C. Cells were again washed with FACS buffer and incubated in a solution containing the secondary antibody Donkey anti-Mouse IgG Alexa Fluor 546 (Invitrogen, #A10036, 1:250) for 30 min at 4°C. Cells were washed a final time prior to flow cytometry analysis.

A BD LSRFortessa was used for flow cytometry analysis of transfected NRVMs. PMT detector voltages were optimized so that all positive signals fell within the linear range. Compensation beads were prepared using AbC™ compensation bead kits according to the manufacturer's instructions. Beads were first incubated with primary antibodies: sarcomeric alpha-actinin (Sigma-Aldrich; #A7811; 1:500) or anti-V5 (Abcam, ab9116, 1:250). After washing, beads were incubated with secondary antibodies: Donkey anti-Mouse IgG Alexa Fluor 546 (Invitrogen, #A10036, 1:250) or Donkey anti-rabbit IgG Alexa Fluor 488 (Invitrogen, #A-21202, 1:250). ArC™ beads were prepared using LIVE/DEAD™ Fixable Blue Dye according to the

manufacturer's instructions. After performing compensation, at least 10,000 events were collected for each sample. Data was analyzed using FlowJo software.

Immunoblotting

Western blots were performed with antibodies against FLAG (Millipore Sigma; #F7425; 1:2000), GAPDH (BioRad; #MCA4739; 1:2000), Connexin-43 (Millipore Sigma; #C6219; 1:1000), and Calnexin (Millipore Sigma; # SAB2501291; 1:2000). Briefly, IVT TBX18 and IVT GFP transfected NRVMs were homogenized in RIPA buffer containing a protease inhibitor cocktail (Sigma). Protein content was quantified by BCA assay and cell lysates (6 µg per lane) were run on a Bolt™ 10% Bis-Tris Plus gel (ThermoScientific; #NW00102BOX) and transferred onto a Immobilon-FL PVDF membrane (Millipore Sigma; #IPFL00010). Then the transferred membrane was incubated with a primary antibody overnight at 4 °C, followed by 1 hour incubation with an appropriate IRDye 800 or 680 secondary antibody (LI-COR; 1:10000). The membrane was then scanned with an Odyssey® CLx Imaging System (LI-COR Biosciences, Lincoln, NE) and analyzed use ImageStudio Lite v5.2.

Immunostaining and Histology

Rat hearts were fixed in 4% PFA followed by dehydration in 30% sucrose. The tissues were embedded and frozen in OCT medium. Frozen 8 µm thick sections were cut using a cryotome and mounted on glass slides. Sections were defrosted, and permeabilized with 0.2% Triton X-100 for 10 minutes at room temperature. Blocking was performed with a blocking solution consisting of 1x PBST containing 5% donkey serum and 1% bovine serum albumin. Slides were then incubated in blocking solution containing the appropriate primary antibodies: sarcomeric alpha-actinin (Sigma-Aldrich; #A7811; 1:800), GFP (ThermoScientific; #A11122; 1:250), CD45 (BioLegend; #202201; 1:250), CD11b/c (BioLegend; #201801; 1:250), CD8a (BioLegend; #201701; 1:250), TBX18 (Abcam; ab115262; 1:200), and Vimentin (Abcam; ab24525; 1:200). Following washes in 1x PBST, slides were incubated in 1x PBST containing the appropriate secondary antibodies: Donkey anti-rabbit IgG Alexa Fluor 488 (Invitrogen, #A-21202, 1:250) and Donkey anti-Mouse IgG Alexa Fluor 546 (Invitrogen, #A10036, 1:250). Additional washes in 1x PBST were performed, and slides were treated with TrueBlack (Biotium) according to manufacturer's instructions to reduce autofluorescence. Slides were stained with DAPI for 10 minutes at room temperature, and mounted with Prolong gold. Tissue sections were imaged on a Zeiss LSM 710 confocal microscope using a 20x 0.8NA Plan-Apochromat objective. Stitching was performed using stitching algorithms in Zen.

Two weeks following direct myocardial injection of naked IVT mRNA in rats (n=3), hearts were extracted for histological examination. Rat hearts were fixed in 10% (v/v) neutral buffered formalin, processed in ethanol and xylene, and embedded in paraffin. Paraffin blocks were sectioned at 8µm thickness with a microtome. Slides were sent to HistoWiz, Inc., Brooklyn, NY, for Masson's Trichrome staining and scanned for imaging. Stained sections of all hearts revealed scar formation at the injection site, marked by blue-stained collagen fibers. Fibrosis border zone was calculated for each section by subtracting the total fibrosis area from the central fibrosis area. Central fibrosis area was defined as containing only blue collagen fibers. Border zone, surrounding the central fibrosis area, contained a mix of red myocytes and blue collagen. Close examination of these slides showed areas of central fibrosis, devoid of red-stained cardiomyocytes, and border zone, mixture of collagen and myocytes (Supplementary Fig. 7d). Accounting for variations in mRNA injected spread and location, we observed a significant degree of fibrosis surrounding cardiomyocytes in the border zone of mRNA TBX18-injected hearts (Supplementary Fig. 7d). Further quantification of the border zone area revealed significantly more fibrosis in mRNA TBX18-injected hearts (Supplementary Fig. 7e). TBX18-induced fibrosis could be inhibited via systemic release of the small molecule A83-01, through an implanted osmotic pump. Rats treated

with a combination of mRNA TBX18 and A83 showed a significant reduction in the level of fibrosis, similar to that seen with control GFP.

Pig hearts were excised at 4-weeks following AV ablation and mRNA injection. Pieces of cardiac tissue were cut from the approximate ablation site and fixed in 10% (v/v) neutral buffered formalin. Selected tissue pieces were then paraffin-embedded and sectioned via microtome. Sister sectioned slides were stained with either Masson's Trichrome or Hematoxylin and Eosin staining solutions and imaged with a Hamamatsu NanoZoomer SQ whole slide scanner.

In situ hybridization
Sections from rat hearts injected with GFP IVT mRNA or TBX18-GFP IVT mRNA were processed for Gja1 mRNA and GFP protein visualization. Endogenous Gja1 mRNA transcripts (ACD 414381-C2) were visualized using RNascope Multiplex Fluorescent Reagent Kit v2 (Advanced Cell Diagnostics 323100) according to manufacturer's instructions. A negative control probe was used to confirm specificity of hybridization. After completing the RNAscope protocol, immunostaining was performed to visualize the expressed GFP protein. Slides were blocked and stained overnight using a rabbit anti-GFP antibody (Thermo A-11122). At a 1:250 dilution. Slides were incubated with donkey anti-rabbit antibody (Thermo A32795) at a 1:250 dilution and counterstained with DAPI prior to mounting with prolong gold reagent. Representative images of GFP-expressing cells were acquired using a Plan-Apo 40x 1.3 NA oil objective on an UltraVIEW Spinning Disk Confocal Microscope equipped with a Hamamatsu Flash 4.0v2 CMOS camera. Images were captured and pre-processed using Volocity Software v7 (PverkinElmer).

Optical mapping
NRVM monolayers were optically mapped with the voltage-sensitive dye, FluoVolt™ (Molecular Probes Invitrogen, #F10488). On the day of recording, NRVM monolayers were loaded with FluoVolt™ as per manufacturer's protocol. Following 20 minutes of incubation of cells with dye, the FluoVolt™ loading solution was washed out twice and cells were imaged in 1x Normal Tyrode's solution (pH 7.35). Cells in Tyrode's solution were kept in a live-cell imaging chamber at 37 degrees Celsius. Using a standard GFP filter set, electrical propagations were recorded as 10 second videos during both spontaneous and electrically stimulated beats. Monolayers were paced using a bipolar, point-source platinum wire connected to a MyoPacer Field Stimulator (IonOptix LLC, Westwood, MA). Square pulse waves were generated at 12V with 5ms duration at 1,2, and 3Hz pacing frequencies. Contour heat maps and conduction velocity measurements were generated using a custom MatLab (MathWorks, Inc., Natick, MA) code, as previously described.[2] Conduction velocities were calculated across an optically mapped 16.6mm diameter area at spontaneous and paced frequencies (1-3Hz).

***In vivo* and *ex vivo* luciferase expression**
At peak vector expression (adenovirus 3 days and IVT mRNA 1 day), animals were sacrificed and imaged to assess biodistribution with immunostaining and IVIS bioluminescence. Heart, liver, lung, spleen, kidney, and lengths of intestine (10 cm immediately distal to the stomach) were harvested from each rat and immediately washed in ice cold PBS. After removal of excess blood, all organs were stored in ice cold DMEM until being incubated with D-luciferin Bright-Glo (Promega; #E2610) for 10 minutes at room temperature. After incubation, corresponding organs from all rats were simultaneously imaged with an IVIS Spectrum (PerkinElmer, Waltham, MA). The total flux of luminescence detected was divided by the respective organ with saline injection (fold-change over saline). Similarly, mice were imaged *in vivo* at specified time points (0 hours to 7 days) to measure the time course of IVT mRNA expression. C57BL mice were equivolume injected with either vehicle (sterile saline) or IVT fLuc mRNA (150 µg/heart) at the LV free wall in a total volume of 50 µL. To visualize luciferase expression, mice were anesthetized with isoflurane

and injected intraperitoneally with D-luciferin Bright-Glo (Promega; #E2610; 150 ug/g body weight). After 10 minutes, mice were imaged with 1 minute exposure in an IVIS Spectrum (Perkin Elmer, Waltham, MA).

Telemetry ECG recording in CAVB rats post-gene delivery

After cardiac injection of IVT mRNA, ECGs were recorded continuously by implanting a dual-lead, biopotential telemeter (TR50BB, Kaha Sciences Inc., Auckland, New Zealand) in the abdominal cavity according to the manufacturer's instructions. Lead I was obtained by positioning the electrodes on the left and right latissimus dorsi muscles. Lead II was obtained by positioning the electrodes between the suprasternal fossi and left lower thoracic cage. The electrical signals were wirelessly transmitted to a SmartPad (TR181, Kaha Sciences Inc., Auckland, New Zealand) and analyzed with LabChart Pro (ADInstruments, Colorado Springs, CO). Telemetry signals were recorded 24/7 over the course of 2 weeks and analyzed to calculate heart rate average and standard deviation for each hour.

Arrhythmia assessment of IVT TBX18

Susceptibility to arrhythmia was assessed for each rat at the completion of the 2-week telemetry recording using a standardized protocol for Programmed Electrical Stimulation (PES). Animals were anesthetized with 5% isoflurane and placed on a mechanical ventilator after intubation. Anesthesia was maintained with 2% isoflurane during PES. A median sternotomy was performed to expose the heart. Positive and negative electrode platinum wires, insulated in a polyethylene tube, were then inserted to the myocardium of the apex. PES was performed with current stimulus isolator (FE180, ADInstruments, Colorado Springs, CO) with or without isoproterenol (3 mg/kg, ip) as done in previous rat arrhythmia studies.[3,4] Pacing thresholds were determined, and stimulation was delivered at a 1 ms pulse width at twice the capture threshold. An S1 drivetrain of 10 stimuli (100 ms interval) was applied followed by an extra stimulus (S2) starting at a coupling interval of 80 ms and by 1 ms decrements until the effective refractory period was reached. Another extra stimulus (S3) followed S2, decreased by 1 ms until the ventricular effective refractory period was reached. After this, a train of programmed stimulation with three extra stimuli after S1 (S2–S4) was performed until a ventricular arrhythmia was induced. If the rat failed to develop ventricular arrhythmia with 3 extra stimuli (S1–S4), the animal was deemed non-inducible.

Optical Mapping and Sharp Electrode Recording

Healthy rat hearts were injected with either GFP or TBX18 mRNA in saline at the left ventricular free wall. A loose suture was tied to mark the site of injection. 3-days following injection, hearts were excised and Langendorff-perfused with 1x Tyrode's solution at 37 degrees Celsius. (±)-Blebbistatin (Cayman Chemical; #13186) was used as an excitation-contraction uncoupler. Hearts were then loaded with voltage-sensitive Di-4-ANEPPS (Invitrogen; #D1199). Fluorescence images of APs were recorded from the anterior surface of the heart using a CMOS camera (100 × 100 pixels, 1000 frames/sec, 1.0 × 1.0 cm2 field of view, Ultima-L, SciMedia, Japan). Activation maps and APD maps were generated using dF/dt for activation and dF2/dt2 for repolarization using digital image analysis routines. Surface electrocardiogram electrodes were positioned surrounding the heart to simultaneously collect electrical potential changes. Following optical mapping recordings, pulled-glass pipettes of 2-3 MΩ resistance were filled with 3M KCl for sharp electrode recordings. Action potential-like signals were acquired at a sampling rate of 20 kHz with Multiclamp 700B microamplifier (Molecular Devices, Union City, CA) and a Digidata 1322 digitizer (Molecular Devices, Union City, CA), and analyzed with pClamp 9.0. Normal Tyrode's solution was used as the extracellular bath solution.

Immune Panel and NRVM qPCR

Transcript levels of either cultured NRVMs or extracted hearts were measured with qRT-PCR. NRVMs were transfected as previously described above with either TBX18 or GFP IVT mRNA. Each rat was injected with either 300ug of naked GFP mRNA, $0.5x10^9$ PFU of GFP Adv, or equivolume saline. mRNA was isolated using Trizol (ThermoFisher Inc.; #15596026), and reverse transcribed to cDNA using PrimeScript™ RT Reagent Kit with gDNA Eraser (Takara Bio Inc.; #RR047A). RT-PCR was carried out using a two-step RT-PCR protocol with the Rotor Gene Q PCR cycler (Qiagen Inc.). Briefly a reaction mix, containing 2x PerfeCTa® SYBR® Green FastMix® (QuantaBio Inc.; #101414-278), 1µM forward and reverse primers for the gene of interest, and nuclease-free water, was prepared. cDNA and reaction mix were aliquoted to respective reaction tubes and mixed well. Forward and reverse SYBR Green KiCqStart™ primers for Hcn4, Kcnj2, Pln, and Rpl4 were purchased from Sigma-Aldrich. Custom innate immune panels were made with TaqMan™ Gene Expression Assays system (Thermo Fisher Inc.). Each reaction was carried out using 3 technical replicates.

Pig CAVB model creation

All animal surgical procedures and care were approved by IACUC of Emory University School of Medicine. Four-month-old female domestic Yorkshire crossbred swine (40-50 kg bodyweight) were enrolled in this study. General anesthesia was induced with ketamine (20 mg/kg), intravenous propofol (4-6 mg/kg), and maintained on isoflurane with endotracheal intubation and positive pressure ventilation. Each animal was placed in the supine position on the table for the procedure. Short sheath for vascular access was inserted in right femoral vein (10-French) and artery (8-French). Location patch for NOGA mapping system was placed on the back side of animal. Twelve-lead ECG patches were placed on the precordial area and limbs. Defibrillation patches were placed on the chest wall.

Screw-type single ventricular pacing lead was inserted through the left internal jugular vein. The pacemaker lead was screwed and fixed to the right ventricular apex under fluoroscopic guidance. A Medtronic permanent pacemaker, programed as VVI 50 BPM, was implanted in the left side neck pocket. Prophylactic lidocaine (40µg/kg/min) was infused for preventing ventricular tachyarrhythmia during RF ablation. Steerable long sheath (Agilis™ NxT, Abbott) was inserted through right femoral venous sheath. RF ablation catheter (Blazer™ XP, 8F/8mm, Boston Scientific) was inserted in this long sheath and placed in the atrioventricular nodal region. RF ablation was performed when intracardiac electrocardiogram showed His-bundle potential and one to one or one to two ratios of atrial and ventricular electrical signal intensity. RF energy setting was 70 Watts and 70°C for 40 seconds. Effective complete AV block was confirmed with 12-lead ECG for 30 minutes.

Porcine 3D intracardiac propagation mapping

A real-time 3D electro-anatomical mapping of the right ventricle was performed with NOGA® XP cardiac navigation system (Johnson and Johnson) before and after atrioventricular node ablation. The 7-French NogaStar® mapping catheter was inserted in the steerable long sheath. Endocardial activation time relative to a reference electrogram was measured from the tricuspid valve anulus to right ventricular apex. The tricuspid anulus was tagged with at least four points (anterior, posterior, medial and lateral). 3D endocardial activation heat maps were generated from previously marked anulus to interventricular septum and right ventricular free wall at >30 point in each area. The earliest activation site was marked in red, while the latest activation area with purple. A final 3D electro-anatomical propagation map of right ventricle was recorded at the end of the study (week 4).

Pig Telemetry implantation

Implantable telemetry (L11, Data Sciences International) was inserted median side of the right sternocleidomastoid muscle. Right internal carotid artery was cut down for pressure gauge lead implantation. The pressure gauge lead was placed in the aortic arch. A subcutaneous tunnel was created with a long trocar from the right side incision pocket for telemeter to the xiphoid process. The apex biopotential lead was secured near the xiphoid process through this tunnel. The intravenous biopotential lead was implanted in the superior vena cava near the sinus node junction via the right internal jugular vein. The intravenous biopotential lead placement was adjusted and fixed when P-wave height reached one-third of the QRS height. Continuous ECG telemetry was recorded for 4 weeks.

Pig Electrophysiological study

Pacemaker interrogation was done each week following implantation. Electrophysiological assessment of ventricular recovery time (VRT) and isoproterenol challenge test (ICT) were performed at 2-weeks and 4-weeks after modified mRNA injection. VRT was measured analogous to sinus node recovery time, clinically used for evaluation of sinus node function. Burst stimulation with 500ms cycle length was delivered via implanted electronic pacemaker for one minute, after which pacing was stopped. VRT was defined as the time interval between last paced beat and first spontaneous beat from the ventricle (Supplementary Fig. 8h). To start ICT, the implanted electronic pacemaker was turned off for measurement of baseline heart late. The electronic pacemaker was turned on only when there was no escape beat, or less than 30 bpm. ICT protocol starts with continuous intravenous infusion of 0.003 μg/min/kg of isoproterenol for five minutes. Infusion concentration was gradually increased to 0.01, 0.03, 0.1 and 0.3μg/min/kg in 5 minute intervals.

Programmed electrical stimulation with and without isoproterenol infusion (0.3μg/min/kg) to assess susceptibility to ventricular arrhythmia induction was performed at week 4. A quadripolar intracardiac pacing catheter was inserted in the right ventricular apex through left femoral venous sheath. Electrical stimulation was started from 10 stimuli (S1) of 500ms interval followed by 1-3 extra stimuli (S2-4). The interval between S1 and S2-4 was progressively decreased by 10ms from 400ms interval to the effective refractory period. Rapid ventricular pacing was started from continuous pacing with 500ms interval that reduced by 10ms in each 10 second until pacing interval reaches to 200ms. If there was no induced ventricular arrhythmia with three extra stimuli and rapid ventricular pacing, the subject was considered non-inducible. A sustained ventricular arrhythmia was defined as any ventricular arrhythmia lasting ≥30 s.

Collagen Gel Contraction Assay

Neonatal rat fibroblasts were freshly isolated from the pre-plates used to enrich cardiomyocytes in the NRVM protocol. On the day of cell isolation, fibroblasts were passaged from the pre-plates onto 60mm tissue culture dishes at 80% confluency (about $3.1*10^6$ cells/dish), and cultured at 37°C for 1-2 hours. Fibroblasts were pre-treated with A83-01 (0.3uM) in 2% FBS-containing F10 media to prevent spontaneous activation. After cells attached, TBX18 or GFP mRNA was transfected at 1ng/k cells for each dish. The next day, successful fibroblast transfection was confirmed by visualization of GFP fluorescence. Cells were then trypsinized and suspended at $0.6\text{-}0.7*10^6$ cells/mL. Transfected fibroblasts were cast in collagen lattice gels at 200,000 cells/gel, using previously published collagen gel contraction assay techniques,[5] with rat tail type-1 collagen (SantaCruz, 3-4mg/mL, sc-136157). Solidified collagen gels were then transferred to individual dishes and cultured over 7-days with 2% FBS media with DMSO or A83-01 (0.3uM). Each gel was imaged with stereoscope each day to track changes in surface area over time.

Statistical Analysis

All quantified data were analyzed for mean, standard deviation (SD), and standard error of mean (SEM). Unless otherwise indicated, data were plotted as mean +/- SEM for all groups. All

quantified bioluminescence total flux data were plotted using a log scale. Due to the variability in injection retention in the myocardium (gene expression found in off-target organs), organ groups contained a non-parametric distribution, and thus a Man-Whitney U test was implemented for comparing luminescence between Adv and IVT vectors. Differences in continuous variables in rats collected over time, such as heart rate, heart rate standard deviation, relative body weight, and echocardiography measurements, were compared using a two-way ANOVA repeated measures with subsequent Tukey's test for multiple comparisons. Data collected with 3 or more groups, such as the immune panel qPCR data set, were evaluated using a one-way ANOVA with Tukey's test for mean comparison. All other data sets were statistically evaluated with a two-sample T test. $P<0.05$ was considered statistically significant for all tests. Statistical analyses were performed using OriginPro 2017 (OriginLab Inc., Northampton, MA).

Supplementary References

1 Kirschman, J. L. *et al.* Characterizing exogenous mRNA delivery, trafficking, cytoplasmic release and RNA-protein correlations at the level of single cells. *Nucleic Acids Res* **45**, e113, doi:10.1093/nar/gkx290 (2017).

2 Park, S. J. *et al.* Insights Into the Pathogenesis of Catecholaminergic Polymorphic Ventricular Tachycardia From Engineered Human Heart Tissue. *Circulation* **140**, 390-404, doi:10.1161/CIRCULATIONAHA.119.039711 (2019).

3 Cho, J. H. *et al.* Delayed Repolarization Underlies Ventricular Arrhythmias in Rats With Heart Failure and Preserved Ejection Fraction. *Circulation* **136**, 2037-2050, doi:10.1161/CIRCULATIONAHA.117.028202 (2017).

4 Lee, T. M., Chen, C. C. & Chang, N. C. Granulocyte colony-stimulating factor increases sympathetic reinnervation and the arrhythmogenic response to programmed electrical stimulation after myocardial infarction in rats. *Am J Physiol Heart Circ Physiol* **297**, H512-522, doi:10.1152/ajpheart.00077.2009 (2009).

5 Ngo, P., Ramalingam, P., Phillips, J. A. & Furuta, G. T. Collagen gel contraction assay. *Methods Mol Biol* **341**, 103-109, doi:10.1385/1-59745-113-4:103 (2006).

Uncropped gels:

Fig. 2i: Western Blot scan

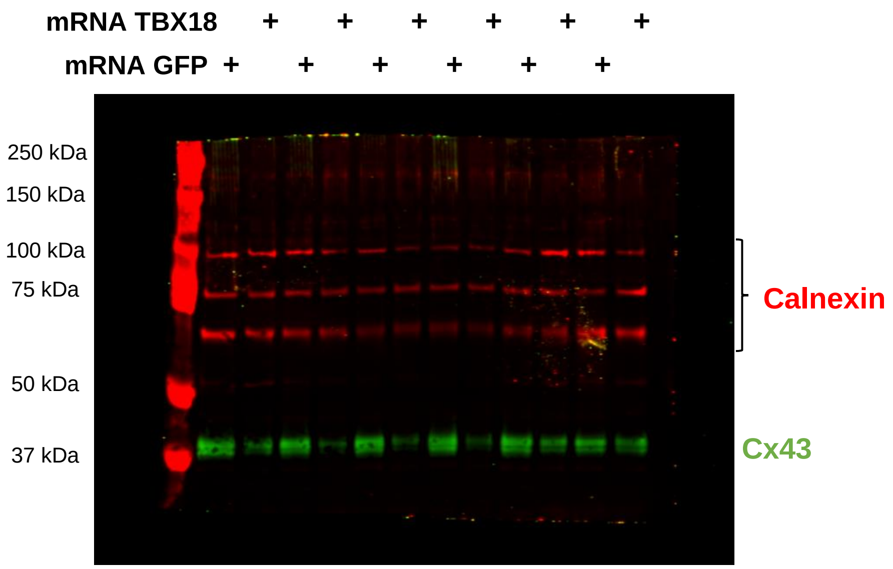

Extended Data Fig. 1b: Western Blot scan

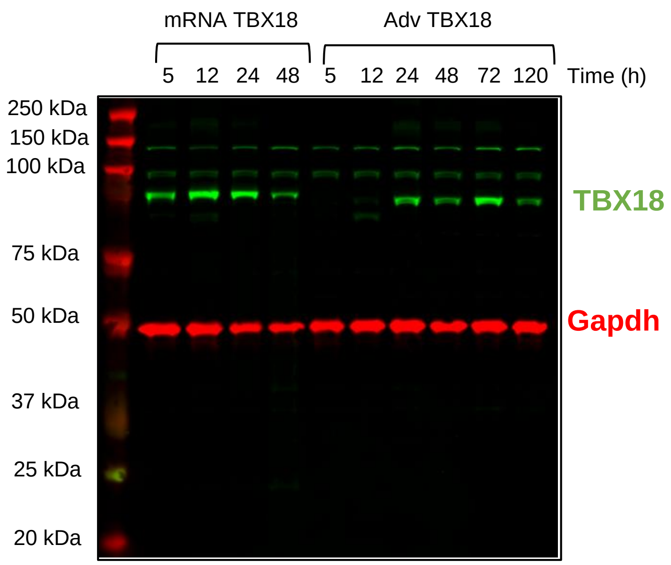

Supplementary Figures:

Supplemental Figure 1

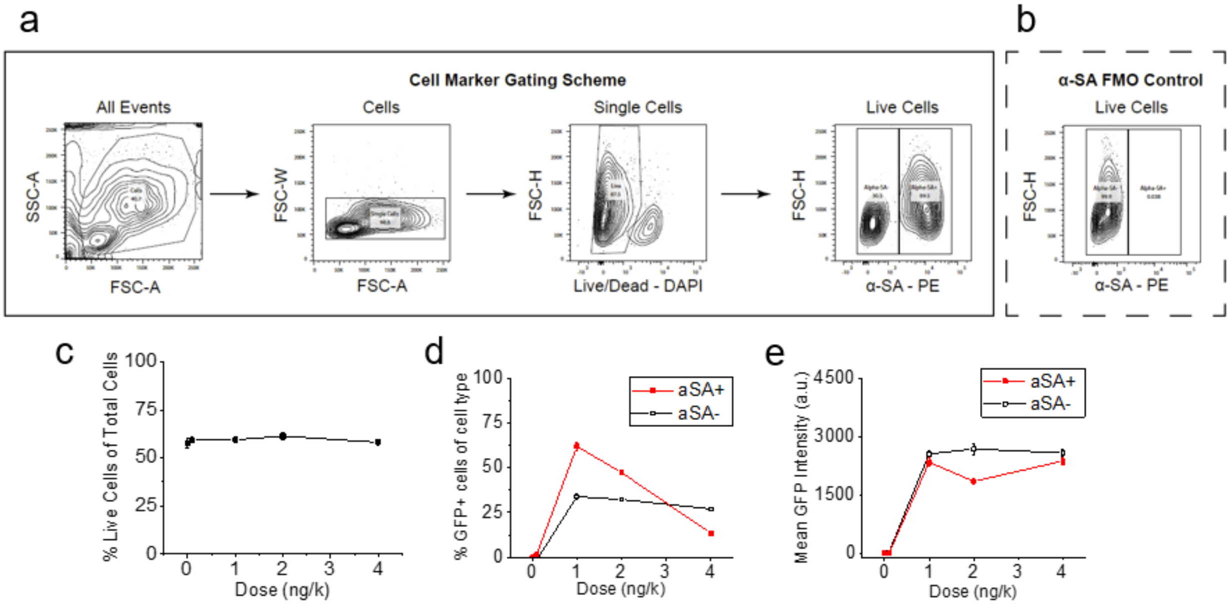

Supplementary Fig. 1: Flow cytometry gating strategy and IVT GFP mRNA dosing in NRVMs. **A**, Flow cytometry gating scheme. All events were gated to isolate single cells, followed by gating dead cells with high DAPI signal, and finally gated by α-sarcomeric actinin to divide myocytes and non-myocytes. **B**, A Fluorescence Minus One (FMO) control for α-sarcomeric actinin staining was included to set the gate for positive myocyte staining. **C**, The proportion of live cells was measured for each transfected dose of mRNA. **D**, Proportion of GFP-expressing myocytes/non-myocytes at various mRNA doses. **E**, Mean GFP-fluorescence intensity in myocytes and non-myocytes at each dose.

Supplemental Figure 2

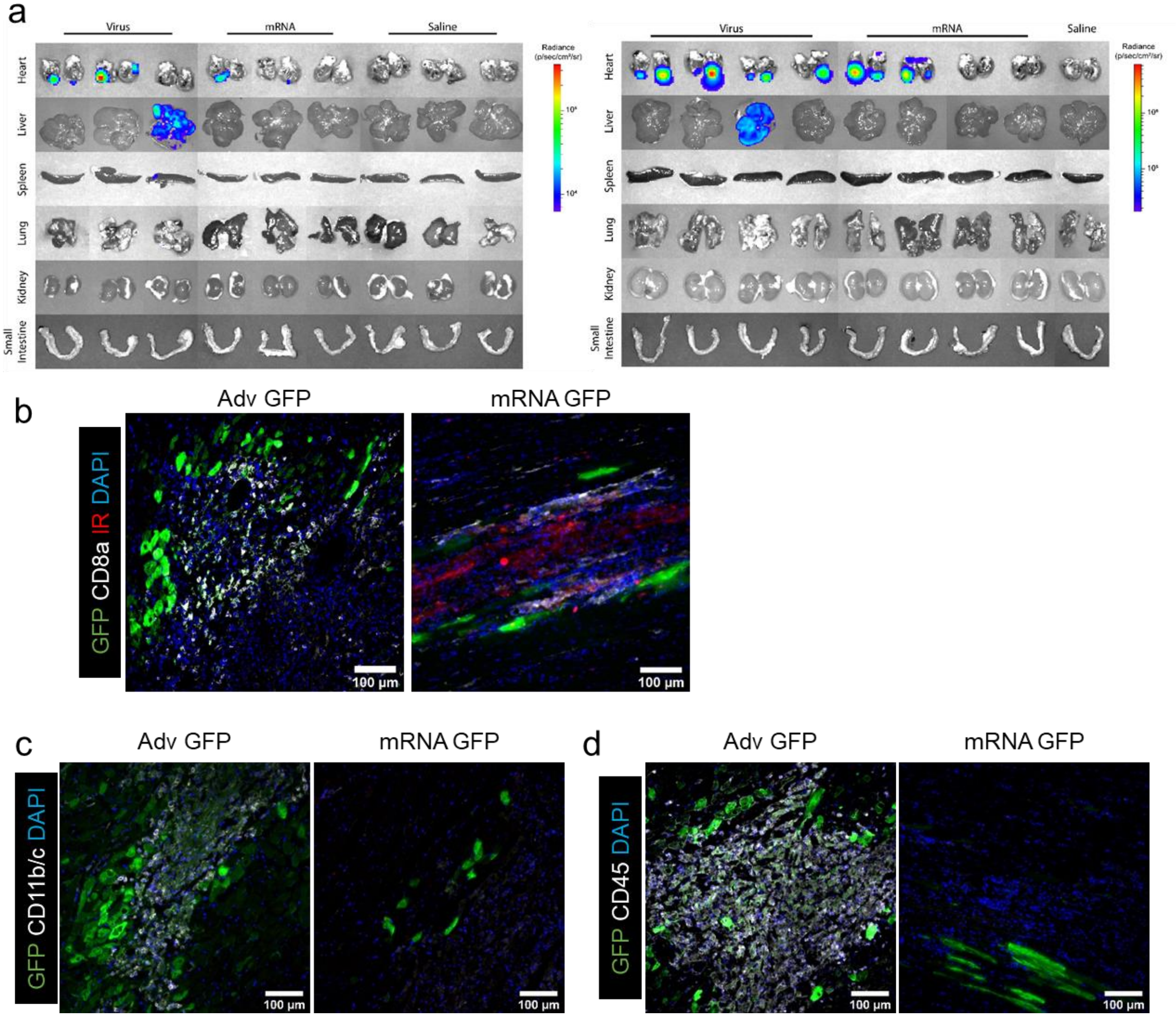

Supplementary Fig. 2: Heart and global biodistribution of IVT mRNA expression *in vivo.*
A, Compiled IVIS images colored by detected luminescence radiance (color bar) for isolated organs in Adv fLuc- (left), mRNA fLuc- (middle), or saline- (right) injected hearts. Results were produced in 2 independent experiments (left & right) and combined for analysis (n=7 rats each).
B-D, Immunostaining for CD8a (**B**), CD11b/c (**C**), and CD45 (**D**) to visualize macrophages near injection sites of Adv GFP- and IVT GFP-injected hearts (n=2). Scale bar, 100µm.

Supplementary Tables:

Table 1. 2-way ANOVA Means Comparisons related to Fig. 2f. IVT mRNA TBX18 vs. fLuc.

Gene	Dose	Gene	Dose	MeanDiff	SEM	q Value	Prob	Alpha	Sig
TBX18	3ng/k	TBX18	1ng/k	-0.08897	0.2108	0.59681	0.97434	0.05	0
fLuc	1ng/k	TBX18	1ng/k	-1.24194	0.2165	8.10909	<0.0001	0.05	1
fLuc	1ng/k	TBX18	3ng/k	-1.15297	0.2165	7.5282	<0.0001	0.05	1
fLuc	3ng/k	TBX18	1ng/k	-1.4944	0.2108	10.0249	<0.0001	0.05	1
fLuc	3ng/k	TBX18	3ng/k	-1.40543	0.2108	9.42809	<0.0001	0.05	1
fLuc	3ng/k	fLuc	1ng/k	-0.25246	0.2165	1.64843	0.65207	0.05	0

Table 2. 2-way ANOVA Means Comparisons related to Fig. 2f. Adv TBX18 vs. Adv mCherry.

Gene	Dose	Gene	Dose	MeanDiff	SEM	q Value	Prob	Alpha	Sig
TBX18	MOI 5	TBX18	MOI 2	0.05461	0.1869	0.41306	0.99117	0.05	0
mChr	MOI 2	TBX18	MOI 2	-1.46867	0.1869	11.1083	<0.0001	0.05	1
mChr	MOI 2	TBX18	MOI 5	-1.52328	0.1869	11.5213	<0.0001	0.05	1
mChr	MOI 5	TBX18	MOI 2	-1.41636	0.1869	10.7127	<0.0001	0.05	1
mChr	MOI 5	TBX18	MOI 5	-1.47098	0.1869	11.1257	<0.0001	0.05	1
mChr	MOI 5	mChr	MOI 2	0.05231	0.1869	0.39562	0.99222	0.05	0

Table 3. 2-way ANOVA Means Comparisons related to Extended Data Fig. 1g. Adv TBX18 vs. Adv mCherry.

Gene	Dose	Gene	Dose	MeanDiff	SEM	q Value	Prob	Alpha	Sig
TBX18	3ng/k	TBX18	1ng/k	-21.6	9.1299	3.3458	0.10274	0.05	0
fLuc	1ng/k	TBX18	1ng/k	-56.4888	9.3801	8.51666	<0.0001	0.05	1
fLuc	1ng/k	TBX18	3ng/k	-34.8888	9.3801	5.26009	0.00372	0.05	1
fLuc	3ng/k	TBX18	1ng/k	-67.6	9.1299	10.4711	<0.0001	0.05	1
fLuc	3ng/k	TBX18	3ng/k	-46	9.1299	7.12532	<0.0001	0.05	1
fLuc	3ng/k	fLuc	1ng/k	-11.1111	9.3801	1.67519	0.64045	0.05	0

Table 4. 2-way ANOVA Means Comparisons related to Extended Data Fig. 1g. IVT mRNA TBX18 vs. fLuc.

Gene	Dose	Gene	Dose	MeanDiff	SEM	q Value	Prob	Alpha	Sig
TBX18	MOI 5	TBX18	MOI 2	-2.3	9.6611	0.33668	0.99517	0.05	0
mChr	MOI 2	TBX18	MOI 2	-68.3	9.6611	9.99789	<0.0001	0.05	1
mChr	MOI 2	TBX18	MOI 5	-66	9.6611	9.66121	<0.0001	0.05	1
mChr	MOI 5	TBX18	MOI 2	-61.1	9.6611	8.94394	<0.0001	0.05	1
mChr	MOI 5	TBX18	MOI 5	-58.8	9.6611	8.60726	<0.0001	0.05	1
mChr	MOI 5	mChr	MOI 2	7.2	9.6611	1.05395	0.87807	0.05	0

Table 5. 2-way ANOVA Means Comparisons related to Extended Data Fig. 6c. IVT mRNA TBX18 vs. GFP +/- A83-01

Gene -Day2	Drug	Gene	Drug	MeanDif	SEM	t Value	Prob	Alpha	Sig
GFP	A83	GFP	DMSO	0.05295	0.0174	3.0278	0.11504	0.05	0
TBX18	DMSO	GFP	DMSO	-0.06613	0.0174	-3.78126	0.04129	0.05	1
TBX18	DMSO	GFP	A83	-0.11908	0.0174	-6.80906	0.00151	0.05	1
TBX18	A83	GFP	DMSO	-0.03308	0.0195	-1.69187	0.80705	0.05	0
TBX18	A83	GFP	A83	-0.08603	0.0195	-4.40002	0.01894	0.05	1
TBX18	A83	TBX18	DMSO	0.03305	0.0195	1.69019	0.80903	0.05	0
**Gene-Day3**	**Drug**	**Gene**	**Drug**	**MeanDif**	**SEM**	**t Value**	**Prob**	**Alpha**	**Sig**
GFP	A83	GFP	DMSO	0.07778	0.0126	6.1702	0.00275	0.05	1
TBX18	DMSO	GFP	DMSO	-0.07678	0.0126	-6.0912	0.00297	0.05	1
TBX18	DMSO	GFP	A83	-0.15456	0.0126	-12.2614	3.30E-5	0.05	1
TBX18	A83	GFP	DMSO	-0.01725	0.0140	-1.22409	1	0.05	0
TBX18	A83	GFP	A83	-0.09503	0.0140	-6.74288	0.0016	0.05	1
TBX18	A83	TBX18	DMSO	0.05953	0.0140	4.22404	0.0235	0.05	1
**Gene-Day4**	**Drug**	**Gene**	**Drug**	**MeanDif**	**SEM**	**t Value**	**Prob**	**Alpha**	**Sig**
GFP	A83	GFP	DMSO	0.1061	0.0139	7.61835	7.46E-04	0.05	1
TBX18	DMSO	GFP	DMSO	-0.07167	0.0139	-5.14614	0.00798	0.05	1
TBX18	DMSO	GFP	A83	-0.17777	0.0139	-12.7645	2.52E-5	0.05	1
TBX18	A83	GFP	DMSO	0.04728	0.0155	3.03611	0.1137	0.05	0
TBX18	A83	GFP	A83	-0.05883	0.0155	-3.77796	0.04146	0.05	1
TBX18	A83	TBX18	DMSO	0.11895	0.0155	7.63895	7.34E-4	0.05	1
**Gene-Day5**	**Drug**	**Gene**	**Drug**	**MeanDif**	**SEM**	**t Value**	**Prob**	**Alpha**	**Sig**
GFP	A83	GFP	DMSO	0.14523	0.0100	14.3880	1.12E-5	0.05	1
TBX18	DMSO	GFP	DMSO	-0.09518	0.0100	-9.42968	1.89E-4	0.05	1
TBX18	DMSO	GFP	A83	-0.24042	0.0100	-23.8177	3.51E-7	0.05	1
TBX18	A83	GFP	DMSO	0.07664	0.0112	6.79118	0.00153	0.05	1
TBX18	A83	GFP	A83	-0.06859	0.0112	-6.07787	0.00301	0.05	1
TBX18	A83	TBX18	DMSO	0.17183	0.0112	15.2253	7.62E-6	0.05	1
**Gene-Day6**	**Drug**	**Gene**	**Drug**	**MeanDif**	**SEM**	**t Value**	**Prob**	**Alpha**	**Sig**
GFP	A83	GFP	DMSO	0.16206	0.0080	20.2364	1.08E-6	0.05	1
TBX18	DMSO	GFP	DMSO	-0.08794	0.0080	-10.9802	6.91E-5	0.05	1
TBX18	DMSO	GFP	A83	-0.25	0.0080	-31.2167	5.36E-8	0.05	1
TBX18	A83	GFP	DMSO	0.11252	0.0089	12.5664	2.80E-5	0.05	1
TBX18	A83	GFP	A83	-0.04955	0.0089	-5.53362	0.00525	0.05	1
TBX18	A83	TBX18	DMSO	0.20045	0.0089	22.3874	5.38E-7	0.05	1
