# Supplementary material for: Transient pacing in pigs with complete heart block via myocardial injection of mRNA coding for the T-box transcription factor 18
Source: Nat Biomed Eng. 2024 May 2;8(9):1124–41. doi: 10.1038/s41551-024-01211-9 (PMC11410671; doi:10.1038/s41551-024-01211-9)
Supplement: Supplementary file 2 — Reporting Summary [file 41551_2024_1211_MOESM2_ESM.pdf]

nature portfolio

Corresponding author(s): Philip Santangelo and Hee Cheol Cho

Last updated by author(s): Jan 4, 2024

# Reporting Summary

Nature Portfolio wishes to improve the reproducibility of the work that we publish. This form provides structure for consistency and transparency in reporting. For further information on Nature Portfolio policies, see our Editorial Policies and the Editorial Policy Checklist.

## Statistics

For all statistical analyses, confirm that the following items are present in the figure legend, table legend, main text, or Methods section.

| n/a | Confirmed | |
|---|---|---|
| ☐ | ☒ | The exact sample size ($n$) for each experimental group/condition, given as a discrete number and unit of measurement |
| ☐ | ☒ | A statement on whether measurements were taken from distinct samples or whether the same sample was measured repeatedly |
| ☐ | ☒ | The statistical test(s) used AND whether they are one- or two-sided<br>*Only common tests should be described solely by name; describe more complex techniques in the Methods section.* |
| ☒ | ☐ | A description of all covariates tested |
| ☒ | ☐ | A description of any assumptions or corrections, such as tests of normality and adjustment for multiple comparisons |
| ☐ | ☒ | A full description of the statistical parameters including central tendency (e.g. means) or other basic estimates (e.g. regression coefficient) AND variation (e.g. standard deviation) or associated estimates of uncertainty (e.g. confidence intervals) |
| ☐ | ☒ | For null hypothesis testing, the test statistic (e.g. $F$, $t$, $r$) with confidence intervals, effect sizes, degrees of freedom and $P$ value noted<br>*Give P values as exact values whenever suitable.* |
| ☒ | ☐ | For Bayesian analysis, information on the choice of priors and Markov chain Monte Carlo settings |
| ☒ | ☐ | For hierarchical and complex designs, identification of the appropriate level for tests and full reporting of outcomes |
| ☐ | ☒ | Estimates of effect sizes (e.g. Cohen's $d$, Pearson's $r$), indicating how they were calculated |

*Our web collection on statistics for biologists contains articles on many of the points above.*

## Software and code

Policy information about availability of computer code

| | |
|---|---|
| Data collection | LabChart 8 Pro, AxIS Axion Biosystems, Leica LASX, Rotor GeneQ, ImageStudio Lite v5.2, FACS diva, Living Image, Fluobeam 700 Near-IR imaging system, Ponemah v6.41, NogaStar mapping (Biosesnse Webster), Nanozoomer Viewer, pClamp11, Volocity software v7. |
| Data analysis | ImageJ 1.52n, FlowJo, LabChart8 Pro, AxIS, Origin Pro 2019, MatLab R2019b, ImageStudio Lite v5.2, FACS diva, LivingImage, Ponemah v6.41, pClamp11, Volocity software v7. |

For manuscripts utilizing custom algorithms or software that are central to the research but not yet described in published literature, software must be made available to editors and reviewers. We strongly encourage code deposition in a community repository (e.g. GitHub). See the Nature Portfolio guidelines for submitting code & software for further information.

## Data

Policy information about availability of data

All manuscripts must include a data availability statement. This statement should provide the following information, where applicable:

- Accession codes, unique identifiers, or web links for publicly available datasets
- A description of any restrictions on data availability
- For clinical datasets or third party data, please ensure that the statement adheres to our policy

The main data supporting the results of this study are available within the paper and its Supplementary Information. All other source data, including the analysed rat

and pig telemetry datasets related to Figs. 5, 7 and 8, which are too large to be shared publicly, are available for research purposes from the corresponding authors on reasonable request.

## Research involving human participants, their data, or biological material

Policy information about studies with human participants or human data. See also policy information about sex, gender (identity/presentation), and sexual orientation and race, ethnicity and racism.

| | |
|---|---|
| Reporting on sex and gender | The study did not involve human participants. |
| Reporting on race, ethnicity, or other socially relevant groupings | – |
| Population characteristics | – |
| Recruitment | – |
| Ethics oversight | – |

Note that full information on the approval of the study protocol must also be provided in the manuscript.

# Field-specific reporting

Please select the one below that is the best fit for your research. If you are not sure, read the appropriate sections before making your selection.

☒ Life sciences ☐ Behavioural & social sciences ☐ Ecological, evolutionary & environmental sciences

For a reference copy of the document with all sections, see nature.com/documents/nr-reporting-summary-flat.pdf

# Life sciences study design

All studies must disclose on these points even when the disclosure is negative.

| | |
|---|---|
| Sample size | Samples size for heart-rate-telemetry studies in rats were constructed to detect an estimated 30% difference between experimental and control groups with a power of 90% and with a two-sided α of 5%. The effect size was estimated on the basis of previously published results related to our work with AdvTBX18 showing heart-rate increases in rat and pig animal models (PMID: 31061413, PMID: 25031269, PMID: 23242162).<br><br>The sample size for functional studies in pigs with complete atrioventricular block was determined on the basis of previously published results by us and others with the same model (PMID: 25031269, PMID: 30947921). The AV-block pig-model data illustrate extreme bradyarrhythmia upon complete atrioventricular ablation, leaving the control, GFP-injected animals nearly fully dependent on the implanted backup pacemaker. Our previous data (PMID: 25031269) showed that a sample size of 4 or higher is sufficient for detecting subjects with mean heart rates that are higher than the backup pacing rate of 50 bpm. We therefore chose to maximize our sample size of TBX18 by conducting our study with n=2 GFP pigs and n=6 TBX18 pigs. |
| Data exclusions | Data from animals who died during surgery or shortly post-operation for AV node ablation were excluded from the study. No other exclusions were used. |
| Replication | All experiments were repeated independently between 2 investigators. Results and data from each experiment were compared to confirm reproducibility. In all cases, we confirmed successful replication of the data reported in the paper. |
| Randomization | All wells (in vitro) and animals (in vivo) were assigned randomly to a treatment group or control group using a random number generator to assign cohorts based on pre-determined sample-size numbers. |
| Blinding | We incorporated a single-blinded study design, in which the operator conducting surgical procedures, data collection, sample preparation, and data analysis was blinded to the treatment group. Upon completion of the study, treatment groups were identified by the unblinded investigator for statistical testing of the data. |

# Reporting for specific materials, systems and methods

We require information from authors about some types of materials, experimental systems and methods used in many studies. Here, indicate whether each material, system or method listed is relevant to your study. If you are not sure if a list item applies to your research, read the appropriate section before selecting a response.

## Materials & experimental systems

| n/a | Involved in the study | |
|---|---|---|
| ☐ | ☒ | Antibodies |
| ☐ | ☒ | Eukaryotic cell lines |
| ☒ | ☐ | Palaeontology and archaeology |
| ☐ | ☒ | Animals and other organisms |
| ☒ | ☐ | Clinical data |
| ☒ | ☐ | Dual use research of concern |
| ☒ | ☐ | Plants |

## Methods

| n/a | Involved in the study | |
|---|---|---|
| ☒ | ☐ | ChIP-seq |
| ☐ | ☒ | Flow cytometry |
| ☒ | ☐ | MRI-based neuroimaging |

# Antibodies

| | |
|---|---|
| Antibodies used | Sarcomeric alpha-actinin (Sigma-Aldrich; #A7811), GFP-Booster (Chromotek, #gba488-100), FLAG (Millipore Sigma; #F7425), GAPDH (BioRad; #MCA4739), Connexin-43 (Millipore Sigma; #C6219), Calnexin (Millipore Sigma; # SAB2501291) GFP (ThermoScientific; #A11122), CD45 (BioLegend; #202201), CD11b/c (BioLegend; #201801), CD8a (BioLegend; #201701), TBX18 (Invitrogen; #PA5-101921), Vimentin (Abcam; #ab24525), V5 tag (Abcam; #ab9116). |
| Validation | Sarcomerica alpha-actinin, FLAG, Connexin-43, TBX18, and Vimentin antibodies were previously validated in-house, using previous rat and mouse heart tissue samples for both WesternBlot and immunostaining imaging. GFP-booster and GFP antibodies were validated for flow cytometry and IF imaging using GFP transfected cells. Calnexin antibody validated with manufacturer's instructions for Western Blot showing a 100kDa band with mouse heart protein lysate. GAPDH antibody validated to react with rat Gapdh protein and Western Blot application by the manufacturer. CD45, CD11b/c, and CD8a antibodies were validated for IF imaging using serial dilutions on PFA-fixed adult rat spleen tissues. |

# Eukaryotic cell lines

Policy information about cell lines and Sex and Gender in Research

| | |
|---|---|
| Cell line source(s) | Primary cells from neonatal rodents, from ATCC (manufacturer). |
| Authentication | Primary cells were previously authenticated by flow cytometry to determine the percentage of alpha sarcomeric actinin-positive cardiomyocytes. HEK293T and HeLa cells were authenticated by the manufacturer (ATCC). |
| Mycoplasma contamination | Cell lines were not tested for mycoplasma contamination. |
| Commonly misidentified lines (See ICLAC register) | No commonly misidentified cell lines were used. |

# Animals and other research organisms

Policy information about studies involving animals; ARRIVE guidelines recommended for reporting animal research, and Sex and Gender in Research

| | |
|---|---|
| Laboratory animals | SAS Sprague Dawley rats: female sex adult (>3 months of age) and all sex neonatal (<3 days old), C57BL/6J mice: all sex adult (>3 months of age) and all sex neonatal (<3 days old), Domestic Yorkshire crossbred pigs: female sex, 4-months old. |
| Wild animals | The study did not involve wild animals. |
| Reporting on sex | All in vitro and staining validation in vivo data were collected from both sexes in an unbiased manner, and analysed together. These data were not analysed separately on the basis of sex as a factor. All in vivo experiments testing biological pacemaker function (in pigs and rats) were conducted in females, owing to their smaller size and higher survival rates after AV-block induction. We do not expect the results of this work to be dependent on gender; thus, experiments designed to test sex differences in heart-rate response after TBX18 delivery were not tested. In future preclinical studies, we will explore the effects of dosing and sex differences further in large animal models. |
| Field-collected samples | The study did not involve samples collected from the field. |
| Ethics oversight | All experiments and procedures involving animals were approved and monitored by the Institutional Animal Care and Use Committee (IACUC) and the Division of Animal Resources (DAR) of Emory University School of Medicine. |

Note that full information on the approval of the study protocol must also be provided in the manuscript.

## Flow Cytometry

### Plots

Confirm that:

☒ The axis labels state the marker and fluorochrome used (e.g. CD4-FITC).

☒ The axis scales are clearly visible. Include numbers along axes only for bottom left plot of group (a 'group' is an analysis of identical markers).

☒ All plots are contour plots with outliers or pseudocolor plots.

☒ A numerical value for number of cells or percentage (with statistics) is provided.

### Methodology

| | |
|---|---|
| Sample preparation | NRVM monolayers were dissociated to a single cell suspension using 0.05% Trypsin-0.53mM EDTA. Suspended cells were stained using LIVE/DEADTM Fixable Blue Dye (Invitrogen) according to the manufacturer's instructions. Cells were then fixed with 4% paraformaldehyde and stored in FACS Buffer (1x PBS + 1% Heat Inactivated FBS) at 4°C until staining was performed at a later timepoint (up to 3 days later). Immediately prior to staining, cells were incubated in Perm/Wash Buffer (BD, 554723) for 20 min at 4°C. Cells were washed with FACS buffer and incubated in a solution containing the primary antibodies sarcomeric alpha-actinin (Sigma-Aldrich; #A7811; 1:4,500) and GFP-Booster (Chromotek, #gba488-100, 1:800) for 30 min at 4°C. Cells were again washed with FACS buffer and incubated in a solution containing the secondary antibody Donkey anti-Mouse IgG Alexa Fluor 546 (Invitrogen, #A10036, 1:250) for 30 min at 4°C. Cells were washed a final time prior to flow-cytometry analysis. |
| Instrument | BD LSR Fortessa |
| Software | FACS Diva (acquisition) and FlowJo (analysis) |
| Cell population abundance | Cells were not live-sorted, and the purity of the sample was not determined. |
| Gating strategy | Cell events were first gated with a FSC-A vs SSC-A plot to remove debris. Single events were gated using a plot of FSC-A vs FSC-W. Live-cell events with dim staining for Thermo Fixable Live Dead Blue dye were gated on Alpha-SA positive, and negative populations were gated using an alpha-SA fluorescence minus one control to set the cutoff. |

☒ Tick this box to confirm that a figure exemplifying the gating strategy is provided in the Supplementary Information.
