# Supplementary figures and images for: Transient pacing in pigs with complete heart block via myocardial injection of mRNA coding for the T-box transcription factor 18
Source: Nat Biomed Eng. 2024 May 2;8(9):1124–41. doi: 10.1038/s41551-024-01211-9 (PMC11410671; doi:10.1038/s41551-024-01211-9)

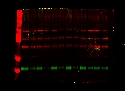

Supplement: Supplementary file 13 — Unprocessed western blots for Fig. 2i. [file 41551_2024_1211_MOESM13_ESM.zip › 0000897_02/0000897_02_TH.jpg]

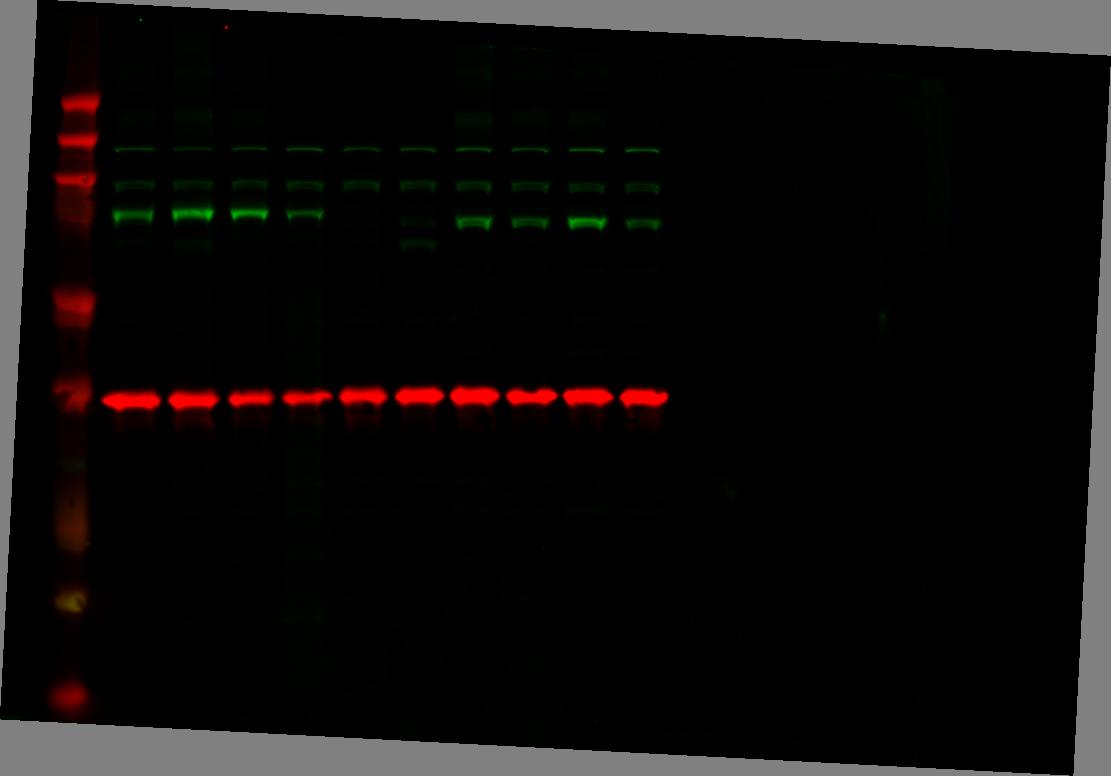

Supplement: Supplementary file 14 — Unprocessed western blots for Extended Data Fig. 1b. [file 41551_2024_1211_MOESM14_ESM.zip › WB_RawFiles_SuppFigure2b/IVTvsAdv_TBX18_Gel4_pic1_06192018.tif.tif]

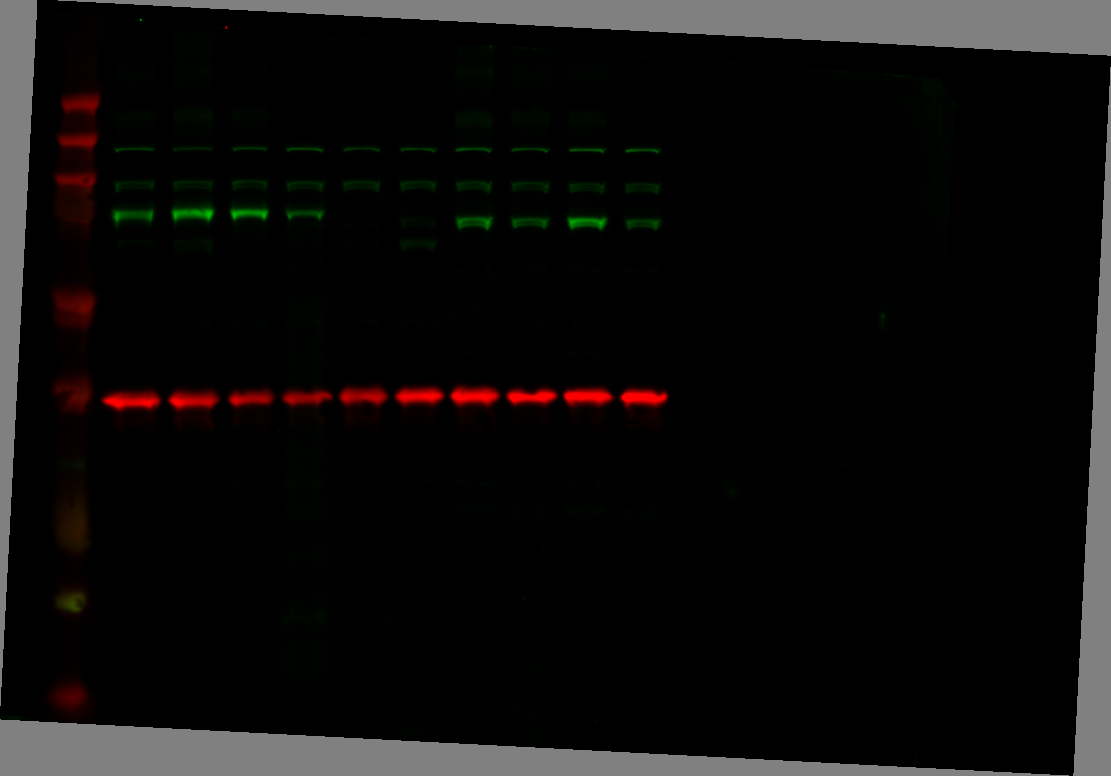

Supplement: Supplementary file 14 — Unprocessed western blots for Extended Data Fig. 1b. [file 41551_2024_1211_MOESM14_ESM.zip › WB_RawFiles_SuppFigure2b/IVTvsAdv_TBX18_Gel4_pic2_06192018.tif.tif]

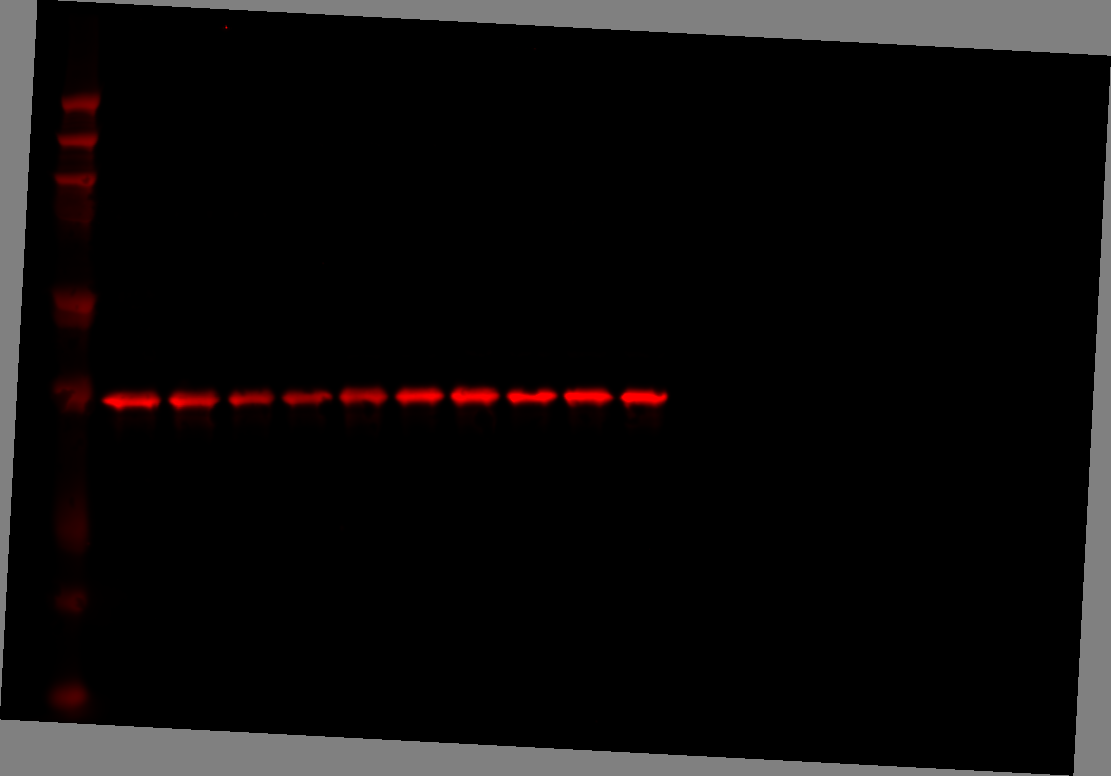

Supplement: Supplementary file 14 — Unprocessed western blots for Extended Data Fig. 1b. [file 41551_2024_1211_MOESM14_ESM.zip › WB_RawFiles_SuppFigure2b/IVTvsAdv_TBX18_Gel4_pic3_06192018.tif.tif]

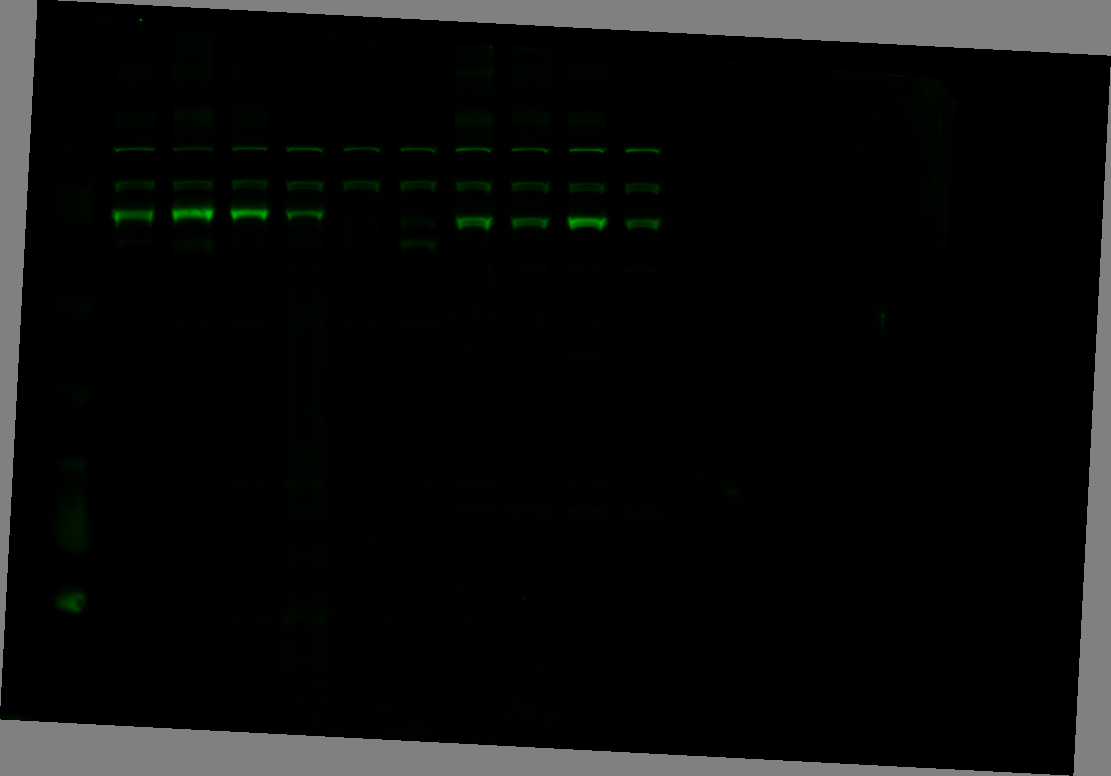

Supplement: Supplementary file 14 — Unprocessed western blots for Extended Data Fig. 1b. [file 41551_2024_1211_MOESM14_ESM.zip › WB_RawFiles_SuppFigure2b/IVTvsAdv_TBX18_Gel4_pic4_06192018.tif.tif]

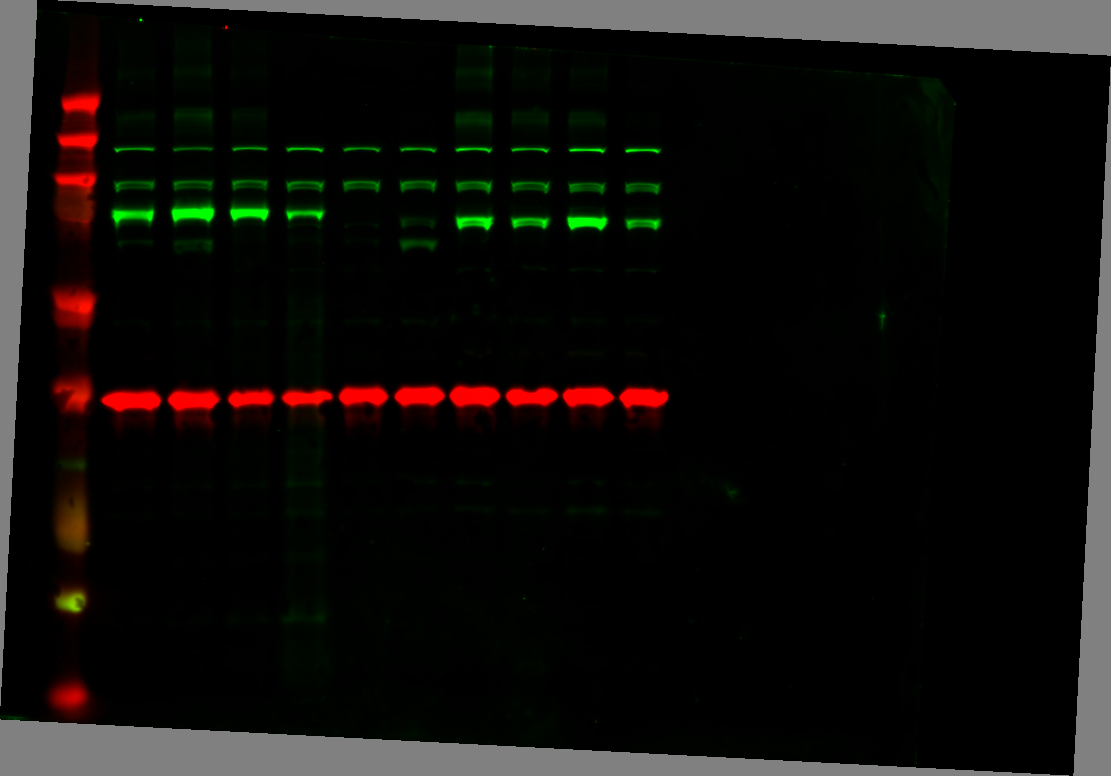

Supplement: Supplementary file 14 — Unprocessed western blots for Extended Data Fig. 1b. [file 41551_2024_1211_MOESM14_ESM.zip › WB_RawFiles_SuppFigure2b/IVTvsAdv_TBX18_Gel4_pic5_06192018.tif.tif]
